# Incremental contribution of Corvis ST dynamic biomechanical parameters to interpretable machine learning prediction of clinician-selected refractive procedures: a retrospective observational study

**DOI:** 10.1186/s12886-026-05208-7

**Published:** 2026-08-20

**Authors:** Yinhao Li, Gang Li, Chuanyun Xu, Jiahui Wang, Xin Wang, Yanli Peng

**Affiliations:** 1 School of Artificial Intelligence, Chongqing University of Technology, Chongqing, 400054 China; 2 School of Computer and Information Science, Chongqing Normal University, Chongqing, 401331 China; 3 Department of Refractive Surgery, Chongqing Aier Eye Hospital, Chongqing, 400020 China; 4 Department of Refractive Surgery, Bright Eye Hospital Group Co., Ltd., Chongqing, 400013 China

**Keywords:** Refractive surgery, Corneal biomechanics, Machine learning, Prediction model, Procedure selection, Artificial intelligence

## Abstract

**Background:**

This study aimed to evaluate whether structured preoperative variables can reproduce clinician-selected refractive procedure patterns and to assess the incremental contribution of Corvis ST dynamic biomechanical parameters beyond conventional refractive, tomographic, pachymetric, and risk-related features.

**Methods:**

We conducted a retrospective observational study of 395 patients (763 eyes) who underwent refractive surgery at Chongqing Aier Eye Hospital between October 2023 and November 2024. The outcome label was the procedure actually selected and performed by clinicians, including ICL, SMILE, LASIK, and SURFACE. Forty-eight structured preoperative features were analyzed, including demographic, refractive, visual, ocular-surface, tomographic, pachymetric, anterior-segment, Pentacam-derived deviation/risk, and Corvis ST-derived variables. Data were split at the patient level into a training cohort and an internal held-out test cohort. Multiple machine learning models were developed using patient-grouped cross-validation, and the final model was evaluated using accuracy, balanced accuracy, macro-F1 score, class-wise metrics, calibration analysis, patient-level clustered bootstrap resampling, and one-eye-per-patient sensitivity analysis. Structured feature-set ablation and SHAP analysis were performed to evaluate feature-domain contributions and model behavior.

**Results:**

The two-stage XGBoost-based model, which first separated ICL from corneal laser procedures and then classified laser procedures into SMILE, SURFACE, and LASIK, achieved the most favorable overall performance. Its estimated total validation accuracy was 83.36% ± 2.59%. On the internal held-out test cohort, the model achieved an end-to-end accuracy of 78.43%, balanced accuracy of 79.17%, macro-F1 score of 77.96%, and macro AUC of 93.77%. Ablation analysis showed that most predictive gain was derived from tomographic and pachymetric variables, whereas pure Corvis ST dynamic biomechanical parameters provided modest complementary information beyond the full non-pure-Corvis feature set. SHAP analysis indicated that refractive parameters were the dominant contributors, while tomographic, pachymetric, risk-related, and Corvis ST-derived variables contributed to procedure-specific model behavior.

**Conclusions:**

Structured preoperative variables can partially reproduce single-center clinician-selected refractive procedure patterns. Corvis ST-derived dynamic biomechanical parameters suggested modest complementary predictive information beyond conventional refractive, tomographic, pachymetric, and risk-related features. These findings support internally validated prediction of clinician-selected procedure patterns but do not establish optimal surgical recommendation or external generalizability.

**Supplementary information:**

The online version contains supplementary material available at 10.1186/s12886-026-05208-7.

## Background

Refractive surgery is widely used to correct refractive errors and restore visual function, with current procedures broadly classified into corneal refractive surgery and phakic intraocular lens implantation, most commonly the implantable collamer lens (ICL) [1]. Corneal refractive procedures, including Small Incision Lenticule Extraction (SMILE), Laser-Assisted In Situ Keratomileusis (LASIK), Laser-Assisted Subepithelial Keratectomy (LASEK), Photorefractive Keratectomy (PRK), and Transepithelial PRK (Trans-PRK), reshape the anterior cornea and are generally safe and predictable, whereas ICL implantation preserves corneal tissue and is often preferred in patients with high myopia, thin corneas, or suspected ectatic risk [2]. Despite favorable safety profiles, inappropriate procedure selection may still result in vision-threatening complications, such as post-operative corneal ectasia following corneal refractive surgery or vault-related anterior segment complications after ICL implantation [3, 4]. Therefore, comprehensive preoperative evaluation incorporating corneal thickness, tomographic patterns, biomechanical characteristics, anterior chamber anatomy, endothelial status, and patient-specific factors is essential for minimizing surgical risk and optimizing refractive outcomes [5].

With the rapid development of artificial intelligence (AI) and machine learning (ML), these techniques have shown substantial promise in refractive surgery [6–12]. A recent narrative review by Choi et al. summarized the expanding applications of AI across both laser vision correction and phakic intraocular lens implantation, including preoperative screening, surgical candidacy assessment, refractive outcome prediction, nomogram optimization, and phakic IOL-related decision-making [13]. In the context of procedure selection and surgical candidacy assessment, Yoo et al. [6] developed multimodal ML classifiers incorporating demographic data, Pentacam tomography, and questionnaire information, achieving excellent AUCs (0.983/0.972) for identifying contraindications. Their subsequent four-class models (LASEK, LASIK, KLEx, and contraindication) achieved accuracies of 81.0% and 78.9%, respectively [8]. However, both studies did not incorporate Corvis ST-derived dynamic biomechanical parameters, leaving their incremental role in procedure-selection pattern prediction insufficiently explored. Li et al. [11] reported 87.75% agreement with surgeons using long-term historical data, but only indirect biomechanical indicators (SRI, SAI, CSI) were available. Overall, although AI has been increasingly applied to refractive surgery screening, candidacy assessment, outcome prediction, and planning, the incremental role of Corvis ST dynamic biomechanical parameters in reproducing clinician-selected procedure patterns across both phakic IOL implantation and corneal laser procedures remains insufficiently explored.

In recent years, corneal biomechanics has become an increasingly important dimension in refractive surgery safety assessment, gaining greater prominence in preoperative screening. The Corvis ST, which is based on dynamic Scheimpflug imaging, quantifies corneal deformation under an air-puff load, and its material-related parameters and composite risk indices have been progressively used to identify subclinical keratoconus and biomechanically vulnerable corneas [14–20]. Moreover, multiple studies have demonstrated that SMILE, LASIK, and surface ablation procedures result in systematic differences in postoperative biomechanical weakening due to variations in ablation depth, flap creation, and tissue-removal patterns [21–26]. From the perspective of residual biomechanical stability, these disparities suggest that different procedures may correspond to different acceptable preoperative risk thresholds: for corneas in a biomechanically borderline state, certain procedures may warrant greater caution or even avoidance, whereas relatively “conservative” procedures may still remain within a safe range. Accordingly, incorporating biomechanical information into procedure selection may provide more comprehensive insights for surgical planning beyond its traditional role in contraindication screening. However, its role in procedure selection remains underexplored.

To address this gap, we conducted a single-center, clinically oriented machine learning study to evaluate whether structured preoperative variables can reproduce clinician-selected refractive procedure patterns across ICL, SMILE, LASIK, and SURFACE. Specifically, we assessed the relative contributions of demographic and refractive variables, Pentacam tomographic and pachymetric parameters, Pentacam-derived deviation and risk indices, composite risk-related indices, and Corvis ST-derived dynamic biomechanical parameters. We further used SHAP analysis to explore how these variables contributed to model behavior in separating ICL from corneal laser procedures and in distinguishing among SMILE, LASIK, and SURFACE. Because the outcome label represented the procedure actually selected and performed by clinicians, the study was designed to analyze single-center procedure-selection patterns rather than to determine an independently verified optimal surgical procedure.

## Methods

### Patients

This single-center retrospective study included patients who underwent refractive surgery at the Refractive Surgery Department of Chongqing Aier Eye Hospital between October 2023 and November 2024. Only patients whose preoperative records were complete were eligible. All the data were anonymized prior to analysis to remove personal identifiers. The study was approved by the Institutional Review Board of Aier Eye Hospital (Approval No. 202401007) and adhered to the tenets of the Declaration of Helsinki.

The study involved four commonly performed refractive procedures: ICL, SMILE, LASIK, and SURFACE. SURFACE included PRK, Trans-PRK, and LASEK. The inclusion and exclusion criteria were established with reference to published guidelines and expert consensus statements, previous literature [27, 28], and the clinical experience of senior refractive surgeons. Eligible patients were 18 to 45 years of age; had completed comprehensive ophthalmic examinations and preoperative assessments; and underwent ICL, SMILE, LASIK, or SURFACE with complete clinical documentation. Eyes with keratoconus or other ectatic corneal disease, visually significant cataracts or glaucoma, active ocular infection or inflammation, severe adnexal abnormalities, retinal pathology affecting visual function, systemic or metabolic disease likely to affect ocular status, or psychiatric conditions impairing cooperation were excluded.

### Clinical procedure selection protocol

Refractive procedure selection was performed according to an institutional clinical workflow based on published guidelines, expert consensus, and the experience of senior refractive surgeons. The following criteria were used as preliminary eligibility and triage rules rather than deterministic decision rules. Final procedure selection was made after comprehensive assessment of ocular findings, estimated tissue-removal safety margins, surgeon judgment, and patient preference.

General criteria for considering refractive surgery included age ≥ 18 years, stable refraction for at least 2 years, intraocular pressure within the normal range, absence of keratoconus or other ectatic corneal disease, absence of visually significant cataract or glaucoma, and no active ocular infection or inflammation. For corneal laser procedures, the refractive range initially considered in our center generally included spherical diopter ≤ −9.00 D, cylindrical diopter ≤ −3.00 D, and spherical equivalent < +6.00 D, provided that corneal tomography, pachymetry, and estimated tissue-removal safety margins were acceptable.

Corneal thickness and tissue-removal reserve were central to the preliminary selection of corneal laser procedures. Stromal laser procedures, including SMILE and LASIK, were generally considered when TCT was ≥ 480 μm and no obvious tomographic or biomechanical risk signals were present. Surface ablation procedures, including PRK, Trans-PRK, and LASEK, could be considered in selected cases with relatively thinner corneas (TCT > 460 μm), particularly when flap creation was not preferred. However, TCT alone was not used as an absolute rule. Final laser-procedure eligibility also considered corneal morphology, anterior and posterior elevation, pachymetric progression, estimated ablation or lenticule depth, residual stromal bed, percent tissue altered, ocular surface status, pupil size, visual demands, and patient preference.

ICL implantation was considered when corneal laser procedures were less suitable, such as in eyes with high myopia, insufficient corneal thickness or tissue-removal reserve, suspicious tomographic-biomechanical findings, large expected tissue removal, or patient preference for a cornea-preserving procedure. ICL eligibility required adequate anterior segment anatomy and safety parameters, including anterior chamber depth, angle status, corneal endothelial status, lens-related parameters, and vault-related assessment based on UBM and AS-OCT examinations.

Because some patients were technically eligible for more than one procedure, the final procedure label reflected clinician-selected treatment under institutional practice patterns rather than a fully objective or universally deterministic decision rule. Procedure selection and surgery were performed by three experienced refractive surgeons at our center, including two chief physicians and one associate chief physician. Although procedures were selected under a shared institutional workflow, routine procedure selection may also be influenced by center-specific and preference-sensitive factors, including available surgical platforms and laser systems, treatment nomograms, cap/flap or lenticule-planning settings, tissue-removal strategies, ICL sizing protocols, surgeon experience and individual surgeon preference, treatment cost, and patient preference. These factors were part of the real-world institutional decision environment but were not fully captured as structured model inputs. Individual surgeon identity was not available in the structured modeling dataset and was not used as a model input feature. Therefore, the procedure labels in this study should be understood as clinician-selected decisions under the equipment, techniques, and clinical workflow of this single center rather than as universal procedure-selection rules or labels derived from purely objective clinical suitability.

TBI and other tomographic-biomechanical indices were reviewed as part of the comprehensive ectasia-risk assessment, but were not used as isolated diagnostic criteria or absolute surgical exclusion thresholds. Eyes with suspected clinical keratoconus or ectatic corneal disease were excluded after comprehensive review by senior refractive surgeons.

### Preoperative examinations and data collection

All enrolled patients underwent standardized preoperative screening, including visual acuity, refraction, intraocular pressure, Pentacam HR (OCULUS), Corvis ST (OCULUS), UBM (SW-3200L), and AS-OCT (Carl Zeiss Meditec) examinations. These examinations were used for routine surgical eligibility assessment and planning. The machine learning model was restricted to consistently available structured preoperative variables.

Among corneal thickness-related variables, TCT derived from Pentacam tomography was included as the primary thickness-related model feature. CCT was not retained as a separate model feature. Procedure-dependent planning parameters, including residual stromal bed, ablation depth, lenticule thickness, and percent tissue altered, were considered during routine clinical planning but were not included as predictors because they depend on the selected or simulated surgical procedure and treatment profile and may introduce post-decision information into procedure prediction.

To improve the interpretability of feature selection and to avoid overattributing tomographic or composite risk-related information to pure biomechanical effects, the included variables were categorized according to both their primary data source and clinical meaning. Specifically, the model inputs were organized into demographic/refractive/visual/ocular-surface baseline characteristics, Pentacam tomographic and pachymetric parameters, Pentacam-derived deviation/risk indices, Corvis ST dynamic corneal response and stiffness-related parameters, and pressure-, thickness-profile-, and composite risk-related indices. A full feature list is provided in Table S1, and the feature domains are summarized in Table 1. Variables describing individual surgeon identity, surgeon-specific preference, detailed patient preference, treatment cost, device or platform availability, specific laser systems, treatment nomograms, cap/flap or lenticule-planning settings, detailed tissue-removal strategy, and center-specific ICL sizing protocol were not available as structured model inputs and were therefore not included in the machine learning model. Consequently, the model was designed to analyze associations between consistently available structured preoperative variables and clinician-selected procedures, rather than to fully reconstruct all institutional, technical, economic, surgeon-specific, and preference-sensitive factors involved in real-world procedure selection.

**Table 1:** Categorization of preoperative variables included in the model

Feature domain	Feature names	Number
Demographic, refractive, visual, and ocular-surface baseline characteristics	Gender, Age, NiBUT, SPH, CYL, Axis, SE, UDVA, CDVA, Dark Pupil Diameter	10
Pentacam tomographic, keratometric, pachymetric, and anterior-segment parameters	Front K1, Front K2, Front K mean, Front K1 Axis, Back K1, Back K2, Back K mean, Back Axis K1, TCT, CV, ACV, ACA, ACD, Front K max, Q Value, Anterior Surface Elevation at TCT, Posterior Surface Elevation at TCT, Pupil Center X, Pupil Center Y, Angle Kappa, CD, IS Value, Average Pachymetric Progression Rate	23
Pentacam-derived ectasia-risk and deviation indices	Df, Db, Dp, Dt, Da, BAD-D, PRFI	7
Corvis ST dynamic corneal response and stiffness-related parameters	SSI, SP-A1, IR, DA-Ratio	4
Pressure-, thickness-profile-, and composite risk-related indices	bIOP, ARTh, CBI, TBI	4

The final dataset comprised 395 patients and 763 eyes. Because both eyes from the same patient are not statistically independent, all model development and evaluation procedures were conducted using patient-level grouping.

The outcome label was the refractive procedure actually selected and performed by clinicians, including ICL, SMILE, LASIK, and SURFACE. Therefore, the modeling task was defined as prediction of clinician-selected procedure patterns rather than identification of an independently verified optimal or safest procedure. Postoperative visual, refractive, biomechanical, and safety outcomes were not used to define the model label.

Available postoperative medical records were reviewed descriptively for documented ectasia-related complications, including postoperative keratoconus progression and corneal ectasia. This review was not used for model training, testing, or outcome definition.

## Model design

**Fig. 1:**
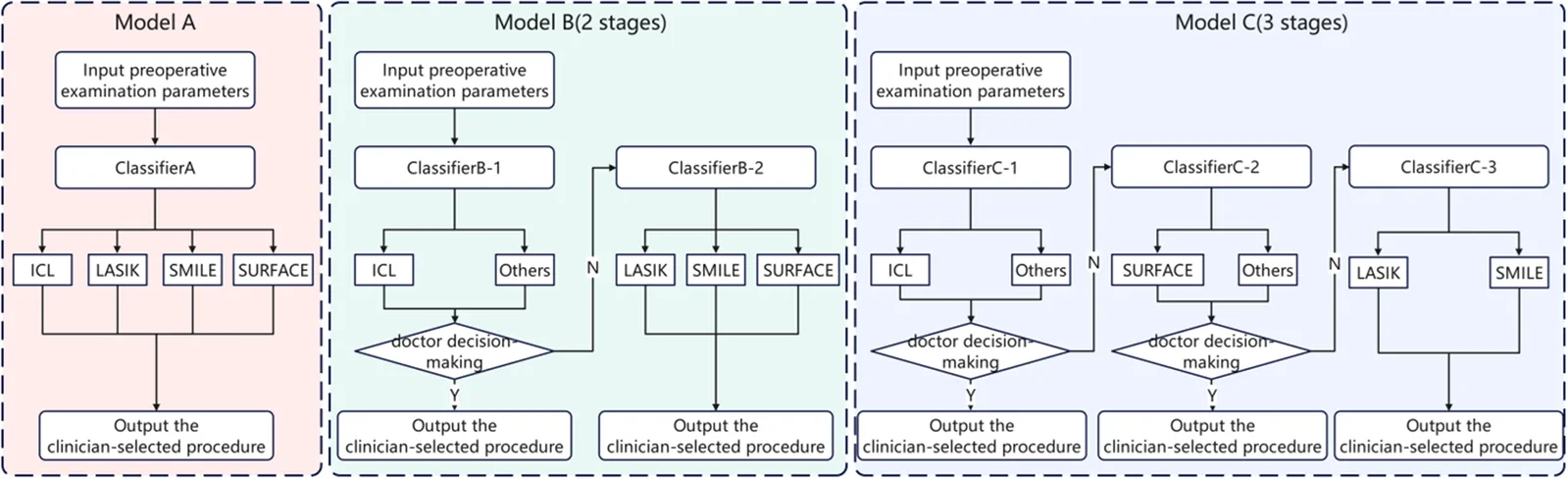
Schematic illustration of the three refractive surgery classification models

To compare different strategies for refractive surgery classification via real-world clinical data, three machine learning frameworks were evaluated (Fig. 1). Model A performed direct four-class classification of ICL, SMILE, LASIK, and SURFACE. Model B used a two-stage hierarchical structure: Stage 1 distinguished ICL from corneal laser procedures, and Stage 2 classified laser procedures into SMILE, LASIK, and SURFACE. Model C extended this hierarchy by adding a third stage: Stage 1 separated ICL from corneal procedures, Stage 2 differentiated SURFACE from stromal procedures, and Stage 3 performed binary classification between SMILE and LASIK.

The multistage designs were clinically motivated task decompositions reflecting major procedural boundaries in refractive surgery selection. The purpose of comparing Models A–C was to determine which task organization provided the most stable internal performance under the current sample size and class distribution.

Six supervised learning algorithms were evaluated: decision tree (DT), random forest (RF), logistic regression (LR), support vector machine (SVM), multilayer perceptron (MLP), and eXtreme gradient boosting (XGBoost).

### Training, validation, and testing procedure

To prevent inter-eye information leakage, all data partitioning was performed strictly at the patient level rather than at the eye level. Thus, both eyes from the same patient were always assigned to the same data partition, and no patient contributed one eye to the training cohort and the fellow eye to the internal held-out test cohort.

The full dataset was split at the patient level using an 8:2 ratio, resulting in a training cohort of 315 patients (610 eyes) and an internal held-out test cohort of 80 patients (153 eyes). Model development was conducted exclusively within the training cohort. Grouped fivefold cross-validation was performed using patient identifiers as grouping labels.

Within each cross-validation fold, preprocessing was performed using the training subset only. These steps included data standardization via Z-score normalization, the removal of highly correlated features using Pearson correlation filtering (threshold = 0.95), and the removal of low-variance features (threshold = 0.015). The fitted preprocessing pipeline was then applied to the corresponding validation subset. SMOTE oversampling was applied only to the training subset within each fold, whereas the validation subset and the internal held-out test cohort remained untouched. Hyperparameter tuning was performed through grid search within the grouped fivefold cross-validation framework.

**Fig. 2:**
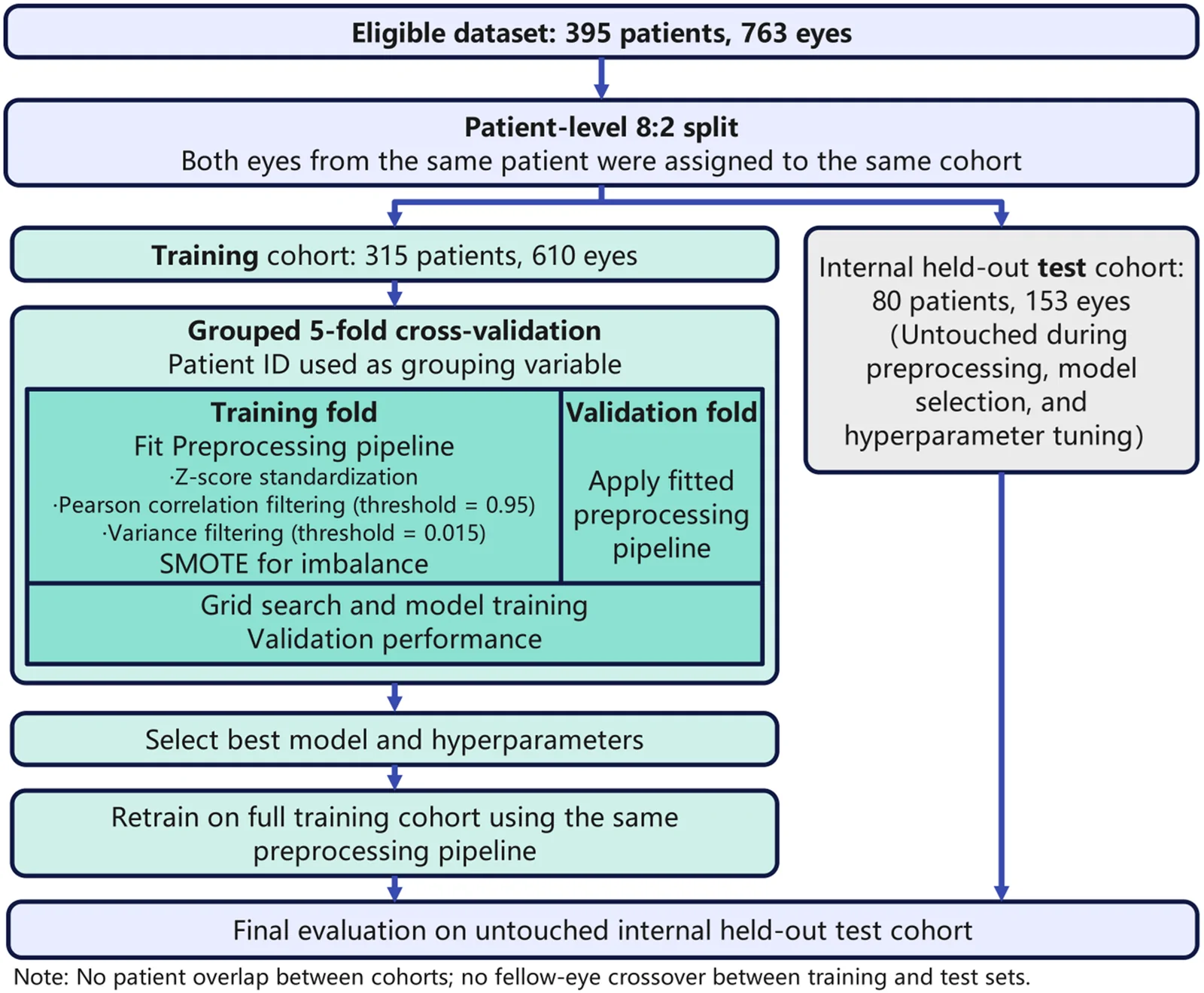
Workflow of patient-level data partitioning, grouped cross-validation, and model development

After model selection, the final model was retrained on the full training cohort using the same preprocessing strategy and evaluated once on the untouched internal held-out test cohort. The complete data partitioning and model development workflow is illustrated in Fig. 2.

The complete hyperparameter grids for the Stage 1 and Stage 2 classifiers are provided in Table S6 and Table S7, respectively. The final selected hyperparameters for the selected Model B classifiers are provided in Table S8. The complete patient-level model development and evaluation procedure is summarized in Algorithm S1. The global random seed was set to 2026 for patient-level data partitioning, cross-validation, model selection, and reproducible analyses whenever applicable. SMOTE oversampling was applied only to the training subset within each cross-validation fold using imbalanced-learn version 0.12.4 with ‘random_state = 7’; all other SMOTE parameters were left as the default settings, including ‘sampling_strategy = “auto”’ and ‘k_neighbors = 5’. Therefore, minority classes in the training subset were oversampled according to the default SMOTE strategy, whereas validation folds and the internal held-out test cohort were never oversampled. The main software environment included Python 3.10, scikit-learn 1.6.1, XGBoost 2.1.4, imbalanced-learn 0.12.4, NumPy 1.26.4, pandas 2.3.2, SciPy 1.13.1, SHAP 0.48.0, and PyTorch 2.0.1+cu118. The structured feature-set ablation analyses used the same patient-level grouping strategy, preprocessing pipeline, SMOTE strategy, random seeds, candidate algorithms, and hyperparameter search procedure as the primary Model B analysis.

## Evaluation metrics

Model performance was evaluated at three levels: Stage 1 classification of ICL versus laser procedures, Stage 2 classification among SMILE, SURFACE, and LASIK, and final end-to-end four-class classification. In addition to overall accuracy, balanced accuracy, macro-averaged precision, macro-averaged sensitivity/recall, macro-averaged specificity, macro-F1 score, weighted metrics, MCC, Cohen’s kappa, and one-vs-rest AUC were reported.

Because class imbalance may make overall accuracy misleading, class-wise sensitivity/recall, specificity, precision/PPV, NPV, F1-score, and one-vs-rest AUC were further reported for each surgical category. Confusion matrices were provided for Stage 1, Stage 2, and the final end-to-end classification.

For each class ${\rm{k}}$, true positives, false positives, false negatives, and true negatives were denoted as ${\rm{T}}{{\rm{P}}_{\rm{k}}}{\rm{, F}}{{\rm{P}}_{\rm{k}}}{\rm{, F}}{{\rm{N}}_{\rm{k}}}{\rm{, and\;T}}{{\rm{N}}_{\rm{k}}}$, respectively. Accuracy was calculated as:

$${\rm{Accuracy = }}\;{{\rm{1}} \over {\rm{N}}}\sum\nolimits_{{\rm{i = 1}}}^{\rm{N}} {{\rm{I}}\left( {{{\rm{y}}_{\rm{i}}}{\rm{ = }}{{{\rm{\hat y}}}_{\rm{i}}}} \right){\rm{,}}} $$

class-wise precision, sensitivity/recall, specificity, and F1-score were calculated as:

$${\rm{Precisio}}{{\rm{n}}_{\rm{k}}}{\rm{ = }}{{{\rm{T}}{{\rm{P}}_{\rm{k}}}} \over {{\rm{T}}{{\rm{P}}_{\rm{k}}}{\rm{ + F}}{{\rm{P}}_{\rm{k}}}}}{\rm{,}}$$

$${\rm{Recal}}{{\rm{l}}_{\rm{k}}}{\rm{/Sensitivit}}{{\rm{y}}_{\rm{k}}}{\rm{ = }}{{{\rm{T}}{{\rm{P}}_{\rm{k}}}} \over {{\rm{T}}{{\rm{P}}_{\rm{k}}}{\rm{ + F}}{{\rm{N}}_{\rm{k}}}}}{\rm{,}}$$

$${\rm{Specificit}}{{\rm{y}}_{\rm{k}}}{\rm{ = }}{{{\rm{T}}{{\rm{N}}_{\rm{k}}}} \over {{\rm{T}}{{\rm{N}}_{\rm{k}}}{\rm{ + F}}{{\rm{P}}_{\rm{k}}}}}{\rm{,}}$$

$${\rm{F}}{{\rm{1}}_{\rm{k}}}{\rm{ = }}{{{\rm{2 \times Precisio}}{{\rm{n}}_{\rm{k}}}{\rm{ \times Recal}}{{\rm{l}}_{\rm{k}}}} \over {{\rm{Precisio}}{{\rm{n}}_{\rm{k}}}{\rm{ + Recal}}{{\rm{l}}_{\rm{k}}}}}{\rm{,}}$$

macro-averaged metrics were calculated as the unweighted mean across classes, whereas weighted metrics were calculated by weighting each class by its support:

$${\rm{Macro - F1 = }}{{\rm{1}} \over {\rm{K}}}\mathop \sum \limits_{{\rm{k = 1}}}^{\rm{K}} {\rm{F}}{{\rm{1}}_{\rm{k}}}{\rm{,}}$$

$${\rm{Weighted - F1 = }}\mathop \sum \limits_{{\rm{k = 1}}}^{\rm{K}} {{{{\rm{n}}_{\rm{k}}}} \over {\rm{N}}} \cdot {\rm{F}}{{\rm{1}}_{\rm{k}}}{\rm{,}}$$

$${\rm{Balanced\;accuracy = }}{{\rm{1}} \over {\rm{K}}}\mathop \sum \limits_{{\rm{k = 1}}}^{\rm{K}} {\rm{Recal}}{{\rm{l}}_{\rm{k}}}{\rm{,}}$$

one-vs-rest AUC was calculated by treating each class as the positive class and all remaining classes as the negative class.

For the internal held-out test cohort and detailed performance analyses, end-to-end performance was calculated directly using the final predicted procedure for each eye. For cross-validation comparison of model architectures, an estimated total validation accuracy was additionally calculated using weighted multiplicative aggregation of stage-wise validation accuracies and class-pathway proportions:

$${\rm{AC}}{{\rm{C}}_{{\rm{Total}}}}{\rm{ = }}\mathop \sum \limits_{{\rm{i = 1}}}^{\rm{n}} \left( {{{\rm{p}}_{\rm{i}}}\mathop \prod \limits_{{\rm{j = 1}}}^{{{\rm{m}}_{\rm{i}}}} {{\rm{a}}_{{\rm{ij}}}}} \right){\rm{,}}$$

where ${\rm{n}}$ represents the number of surgical categories, ${{\rm{p}}_{\rm{i}}}$ represents the proportion of class i in the true dataset, ${{\rm{a}}_{{\rm{ij}}}}$ represents the validation accuracy of class i at decision stage j, and ${{\rm{m}}_{\rm{i}}}{\rm{ }}$ represents the number of stages required by class i For Model B, ICL eyes passed only Stage 1, whereas SMILE, SURFACE, and LASIK eyes passed both Stage 1 and Stage 2. For Model C, ICL eyes passed only Stage 1, SURFACE eyes passed Stages 1 and 2, and SMILE and LASIK eyes passed Stages 1–3. This estimated value was used only for architecture-level comparison during model selection and should be distinguished from the directly calculated end-to-end performance on the internal held-out test cohort.

Because refractive procedure selection is performed at the eye level, the primary model evaluation was conducted at the eye level. However, because fellow eyes from the same patient are not statistically independent, additional analyses were performed to account for inter-eye correlation. Patient-level clustered bootstrap resampling was used to estimate 95% confidence intervals. In each bootstrap replicate, patients rather than individual eyes were resampled with replacement, and all available eyes from each selected patient were retained. A one-eye-per-patient sensitivity analysis was also performed by randomly selecting one eye from each patient in the internal held-out test cohort and repeating this procedure 1,000 times.

Calibration analysis was performed using calibration curves, Brier score, and ECE. Calibration was evaluated for Stage 1, Stage 2, and the final end-to-end four-class output. For multiclass outputs, one-vs-rest calibration was calculated for each surgical category, and macro one-vs-rest Brier score and macro one-vs-rest ECE were reported. Top-label ECE was additionally calculated to assess whether the confidence assigned to the predicted class was consistent with observed correctness. Calibration was assessed on the internal held-out test cohort without fitting any additional calibration model. The multiclass Brier score was calculated as:

$${\rm{Brier = }}{{\rm{1}} \over {\rm{N}}}\mathop \sum \limits_{{\rm{i = 1}}}^{\rm{N}} \mathop \sum \limits_{{\rm{k = 1}}}^{\rm{K}} {\left( {{{\rm{p}}_{{\rm{ik}}}}{\rm{ - }}{{\rm{y}}_{{\rm{ik}}}}} \right)^{\rm{2}}}{\rm{,}}$$

where ${{\rm{p}}_{{\rm{ik}}}}$ is the predicted probability for class ${\rm{k}}$, and ${{\rm{y}}_{{\rm{ik}}}}$ is the one-hot encoded true label. Expected calibration error (ECE) was calculated by grouping predictions into probability bins:

$${\rm{ECE = }}\mathop \sum \limits_{{\rm{b = 1}}}^{\rm{B}} {{{\rm{|}}{{\rm{B}}_{\rm{b}}}{\rm{|}}} \over {\rm{N}}}\left| {{\rm{acc(}}{{\rm{B}}_{\rm{b}}}{\rm{) - conf(}}{{\rm{B}}_{\rm{b}}}{\rm{)}}} \right|{\rm{,}}$$

where ${\rm{acc(}}{{\rm{B}}_{\rm{b}}}{\rm{)}}$ and ${\rm{conf(}}{{\rm{B}}_{\rm{b}}}{\rm{)}}$ represent the observed accuracy and mean predicted confidence in bin ${{\rm{B}}_{\rm{b}}}$, respectively.

### Statistical analysis

The Shapiro–Wilk test was used to assess the distribution of continuous variables. Continuous variables are reported as means ± standard deviations or medians with interquartile ranges, as appropriate. Because several variables violated normality assumptions, intergroup comparisons of preoperative variables among the four clinician-selected procedure groups were performed using the Kruskal–Wallis test. These analyses were used to describe baseline distributional differences across groups and were not interpreted as causal evidence of procedure selection. Statistical significance was defined as a two-sided *p* value < 0.05.

To further evaluate potential redundancy between Corvis ST dynamic biomechanical parameters and tomographic or pachymetric variables, Spearman correlation analysis was performed between pure Corvis ST dynamic response/stiffness parameters and Pentacam tomographic, pachymetric, deviation/risk, and composite risk-related variables. Pure Corvis ST dynamic response/stiffness parameters included SSI, SP-A1, IR, and DA-Ratio. Spearman correlation was used because several preoperative variables were not normally distributed. *p* values were adjusted using the Benjamini–Hochberg false discovery rate method. This analysis was used descriptively to assess information overlap and to contextualize the structured feature-set ablation results.

## Results

### Study cohort and data partition

A total of 763 eyes from 395 patients were included in this study, comprising bilateral cases with identical procedures, bilateral cases with different procedures, and unilateral cases. Among them, 27 records were single-eye cases. To account for the non-independence of fellow eyes, data partitioning was performed at the patient level. The final 8:2 split included 315 patients/610 eyes in the training cohort and 80 patients/153 eyes in the internal held-out test cohort. The patient-level and eye-level distributions across surgical procedures and data partitions are presented in Table 2. Because a small number of patients underwent different procedures in fellow eyes, the sum of procedure-specific patient counts may exceed the total number of unique patients in each cohort.

**Table 2:** Patient-level data partition and eye-level distribution of surgical procedures

Cohort	Patients/Eyes	ICL patients/eyes	SMILE patients/eyes	SURFACE patients/eyes	LASIK patients/eyes
Overall dataset	395/763	100/199	122/218	57/108	126/238
Training cohort	315/610	81/162	99/178	44/85	97/185
Internal held-out test cohort	80/153	19/37	23/40	13/23	29/53

### Baseline characteristics

Normality testing showed that several variables were not normally distributed; therefore, intergroup comparisons of baseline and preoperative variables were performed using the Kruskal–Wallis test. Detailed results for demographic, refractive, visual, tomographic, pachymetric, anterior-segment, and Corvis ST-derived variables are provided in Table S2. Because the outcome label represented clinician-selected procedures rather than randomized treatment allocation, intergroup differences were expected and should be interpreted as descriptive characteristics of historical procedure-selection patterns rather than causal determinants of procedure choice.

Given the clinical relevance of TBI in ectasia-risk assessment, its distribution was further examined across surgical groups. The median TBI values were 0.45 [0.34, 0.59] in the ICL group, 0.33 [0.09, 0.42] in the SMILE group, 0.46 [0.38, 0.59] in the SURFACE group, and 0.31 [0.16, 0.43] in the LASIK group. Relatively higher TBI values were observed in the ICL and SURFACE groups.

In the descriptive review of available postoperative medical records, no postoperative keratoconus progression or corneal ectasia was documented. However, because postoperative follow-up was not standardized as a long-term safety endpoint in this retrospective study, this observation should be interpreted descriptively rather than as evidence of comparative procedural safety.

### Comparison of model architectures

Model architecture and algorithm selection were performed exclusively within the training cohort using patient-grouped fivefold cross-validation. The internal held-out test cohort was not used for preprocessing, feature selection, hyperparameter tuning, algorithm selection, or architecture selection. After model selection, the pre-specified final model was retrained on the full training cohort and evaluated once on the internal held-out test cohort. Table 3 summarizes the cross-validation results used for model selection and the corresponding post-selection held-out test performance. Detailed cross-validation results for all candidate algorithms across the three architectures are provided in Table S3. As supplementary evidence for algorithm comparison within the training cohort, cross-validation ROC curves of the six candidate algorithms used in the two stages of Model B are provided in Figure S1.

**Table 3:** Comparison of model architectures using patient-grouped cross-validation and internal held-out test performance

Model	Selected Algorithm	Stage 1 CV accuracy	Stage 2 CV accuracy	Stage 3 CV accuracy	Estimated total validation accuracy	Test accuracy	Test balanced accuracy	Test macro-F1
A	XGBoost	77.70 ± 3.34	/	/	77.70 ± 3.34	72.55	74.15	72.89
B	XGBoost	93.64 ± 1.60	85.05 ± 3.15	/	**83.36 ± 2.59**	**78.43**	**79.17**	**77.96**
C	XGBoost	93.64 ± 1.60	94.90 ± 2.53	86.49 ± 3.52	82.99 ± 2.81	75.81	76.20	75.68

Values are presented as percentages. Cross-validation accuracies are reported as mean ± standard deviation across patient-grouped fivefold cross-validation folds within the training cohort. The estimated total validation accuracy was calculated using weighted multiplicative aggregation based on class-specific decision pathways and was used for architecture-level comparison. Architecture and algorithm selection were based only on training-cohort cross-validation. Internal held-out test performance was calculated directly from final end-to-end predicted labels and is reported as post-selection evaluation; it was not used for model selection

In training-cohort cross-validation, Model A, which performed direct four-class classification, achieved an estimated total validation accuracy of 77.70% ± 3.34%. Model B, which first distinguished ICL from corneal laser procedures and then classified laser procedures into SMILE, SURFACE, and LASIK, achieved Stage 1 and Stage 2 cross-validation accuracies of 93.64% ± 1.60% and 85.05% ± 3.15%, respectively, with the highest estimated total validation accuracy of 83.36% ± 2.59%. Model C achieved high stage-wise validation accuracies, including 94.90% ± 2.53% in Stage 2 and 86.49% ± 3.52% in Stage 3, but its estimated total validation accuracy was 82.99% ± 2.81%.

Based on these training-cohort cross-validation results, the XGBoost-based Model B was selected as the final architecture because it achieved the highest estimated total validation accuracy while maintaining a clinically interpretable two-stage structure. After this selection step, Model B achieved an end-to-end test accuracy of 78.43%, balanced accuracy of 79.17%, and macro-F1 score of 77.96% on the internal held-out test cohort. The post-selection held-out test performances of the other architectures are also reported in Table 3 for transparency but were not used for model selection.

### Performance of the selected model B

In the internal held-out test cohort, Model B was evaluated at three levels: Stage 1, Stage 2, and final end-to-end four-class classification. In Stage 1, the model achieved an accuracy of 89.54%, balanced accuracy of 83.90%, macro-F1 score of 85.18%, weighted-F1 score of 89.33%, and macro one-vs-rest AUC of 92.57%. In Stage 2, which was evaluated among laser-procedure eyes, the model achieved an accuracy of 82.76%, balanced accuracy of 83.12%, macro-F1 score of 81.65%, weighted-F1 score of 83.03%, and macro one-vs-rest AUC of 94.90%. For the final end-to-end four-class task, the model achieved an accuracy of 78.43%, balanced accuracy of 79.17%, macro-F1 score of 77.96%, weighted-F1 score of 78.43%, and macro one-vs-rest AUC of 93.77% (Table 4).

**Table 4:** Overall performance of model B on the internal held-out test cohort

Task	n	Accuracy	Balanced accuracy	Macro precision	Macro-F1	Weighted-F1	MCC	Cohen’s kappa	Macro AUC
Stage 1: ICL vs Laser	153	89.54	83.90	86.74	85.18	89.33	70.59	70.39	92.57
Stage 2: SMILE vs SURFACE vs LASIK	116	82.76	83.12	81.20	81.65	83.03	73.87	73.34	94.90
Total: End-to-end four-class	153	78.43	79.17	77.64	77.96	78.43	71.02	70.76	93.77

Class-wise analysis further demonstrated heterogeneous performance across surgical categories (Table 5). In the final four-class classification, the model achieved sensitivity/recall values of 72.97% for ICL, 87.50% for SMILE, 82.61% for SURFACE, and 73.58% for LASIK. The corresponding F1-scores were 77.14%, 81.40%, 74.51%, and 78.79%, respectively. Although SURFACE was the smallest class in the internal held-out test cohort, with 23 eyes, its recall remained 82.61%; however, its precision was relatively lower at 67.86%, indicating that some non-SURFACE eyes were misclassified as SURFACE. These findings suggest that overall accuracy alone would not fully capture category-specific performance differences.

**Table 5:** Class-wise performance of model B on the internal held-out test cohort

Task	Class	Support	Sensitivity/Recall	Specificity	Precision/PPV	NPV	F1-score	AUC
Stage 1	ICL	37	72.97	94.83	81.82	91.67	77.14	92.57
Stage 1	Laser	116	94.83	72.97	91.67	81.82	93.22	92.57
Stage 2	SMILE	40	87.50	86.84	77.78	92.96	82.35	93.98
Stage 2	SURFACE	23	82.61	91.40	70.37	95.51	76.00	95.56
Stage 2	LASIK	53	79.25	96.83	95.45	84.72	86.60	95.15
End-to-end	ICL	37	72.97	94.83	81.82	91.67	77.14	92.57
End-to-end	SMILE	40	87.50	90.27	76.09	95.33	81.40	95.07
End-to-end	SURFACE	23	82.61	93.08	67.86	96.80	74.51	94.92
End-to-end	LASIK	53	73.58	93.00	84.78	86.92	78.79	92.55

**Fig. 3:**
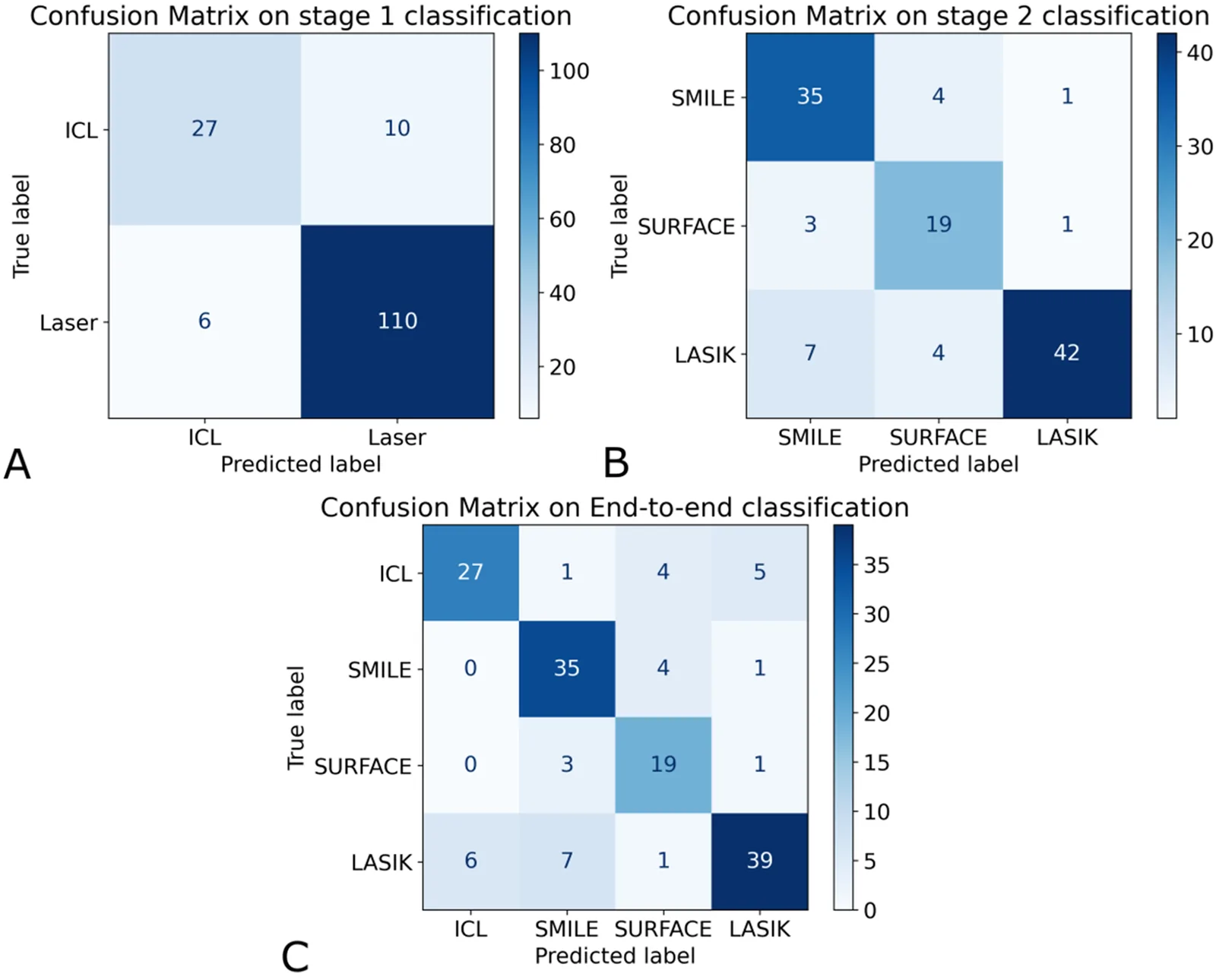
Confusion matrices of model B on the internal held-out test cohort. (**A**) Stage 1 classification of ICL versus laser procedures. (**B**) Stage 2 classification among SMILE, SURFACE, and LASIK among laser-procedure eyes. (**C**) Final end-to-end four-class classification across ICL, SMILE, SURFACE, and LASIK. Rows indicate the true labels, and columns indicate the predicted labels

The confusion matrices are shown in Fig. 3. In Stage 1, 27 of 37 ICL eyes and 110 of 116 laser-procedure eyes were correctly classified. In Stage 2, 35 of 40 SMILE eyes, 19 of 23 SURFACE eyes, and 42 of 53 LASIK eyes were correctly classified. In the final end-to-end four-class classification, the model correctly classified 27 ICL eyes, 35 SMILE eyes, 19 SURFACE eyes, and 39 LASIK eyes. Misclassifications occurred mainly along clinically overlapping procedure-selection boundaries, including ICL versus corneal laser procedures and among laser subtypes.

### Patient-level uncertainty, sensitivity, and calibration analyses

To account for the non-independence of bilateral eyes, patient-level clustered bootstrap analysis was performed (Table 6). For the final end-to-end four-class classification, the bootstrap-derived 95% confidence intervals were 70.70%–85.06% for accuracy, 71.37%–86.31% for balanced accuracy, 69.25%–84.60% for macro-F1 score, 70.63%–85.20% for weighted-F1 score, and 90.74%–96.45% for macro one-vs-rest AUC.

**Table 6:** Patient-level uncertainty analysis and one-eye-per-patient sensitivity analysis

Task	Analysis	Accuracy	Balanced accuracy	Macro-F1	Weighted-F1	Macro AUC
Stage 1	Primary eye-level estimate	89.54	83.90	85.18	89.33	92.57
Stage 1	Patient-clustered bootstrap 95% CI	83.01–95.27	73.45–92.04	75.36–92.48	82.24–95.20	85.84–97.76
Stage 1	One-eye-per-patient mean	89.28	83.49	84.43	89.11	92.23
Stage 2	Primary eye-level estimate	82.76	83.12	81.65	83.03	94.90
Stage 2	Patient-clustered bootstrap 95% CI	74.78–90.00	75.05–90.81	72.26–89.35	75.53–90.09	91.27–97.66
Stage 2	One-eye-per-patient mean	82.28	82.95	81.35	82.57	94.24
Total	Primary eye-level estimate	78.43	79.17	77.96	78.43	93.77
Total	Patient-clustered bootstrap 95% CI	70.70–85.06	71.37–86.31	69.25–84.60	70.63–85.20	90.74–96.45
Total	One-eye-per-patient mean	78.18	79.01	77.83	78.14	93.13

In the one-eye-per-patient sensitivity analysis, the model yielded similar performance estimates, with a mean accuracy of 78.18%, balanced accuracy of 79.01%, macro-F1 score of 77.83%, weighted-F1 score of 78.14%, and macro one-vs-rest AUC of 93.13%. These results suggest that the primary eye-level performance estimates were not materially inflated by the inclusion of bilateral eyes.

Calibration analysis was performed to evaluate the reliability of predicted probabilities beyond discrimination performance. As shown in Table 7, Stage 1 achieved a top-label ECE of 0.0494 and a macro one-vs-rest ECE of 0.0683. Stage 2 showed a top-label ECE of 0.0806 and a macro one-vs-rest ECE of 0.0711. For the final end-to-end four-class output, the top-label ECE was 0.0663, and the macro one-vs-rest ECE was 0.0644. The corresponding calibration curves are shown in Figure S2, and class-wise one-vs-rest calibration metrics are provided in Table S4. Overall, the calibration metrics suggested generally reasonable but imperfect probability calibration, with visible fluctuations in class-wise calibration curves, particularly in smaller categories.

**Table 7:** Calibration performance of model B on the internal held-out test cohort

Task	Multiclass Brier score	Top-label ECE	Macro OvR Brier score	Macro OvR ECE
Stage 1: ICL vs Laser	0.1465	0.0494	0.0732	0.0683
Stage 2: SMILE vs SURFACE vs LASIK	0.2723	0.0806	0.0908	0.0711
End-to-end four-class	0.3219	0.0663	0.0805	0.0644

Brier score and expected calibration error (ECE) were used to evaluate probability calibration. For multiclass outputs, one-vs-rest calibration was calculated for each class, and macro one-vs-rest values were averaged across classes. Top-label ECE evaluated whether the confidence of the predicted class was consistent with observed correctness. Lower values indicate better calibration. Because multiclass Brier score is affected by the number of classes, values should be interpreted within each task rather than directly compared across binary, three-class, and four-class tasks

### Ablation analysis of structured feature domains

To evaluate structured feature-domain contributions, ablation analysis was performed using predefined feature sets. For conciseness, stage-wise cross-validation metrics in Table 8 are reported as the mean performance across six candidate algorithms, whereas algorithm-specific cross-validation results are provided in Table S5. The Test ACC column denotes the end-to-end four-class accuracy of the selected XGBoost-based Model B on the internal held-out test cohort under each feature set. The demographic/refractive baseline model showed limited end-to-end test accuracy of 43.79%. Adding Pentacam tomographic, keratometric, pachymetric, and anterior-segment parameters substantially improved the test accuracy to 69.93%, indicating that corneal morphology and thickness-related variables were major contributors to clinician-selected procedure prediction.

**Table 8:** Ablation analysis of structured feature sets

Model	Feature set	Stage 1 ACC	Stage 1 AUC	Stage 1 Recall	Stage 1 F1-score	Stage 2 ACC	Stage 2 F1-score	Test ACC
M1	Demographic + refractive baseline	75.78	83.00	75.33	75.65	63.36	62.13	43.79
M2	M1 + Pentacam tomography/pachymetry	84.86	90.68	82.14	84.89	73.66	73.37	69.93
M3	M2 + Pentacam BAD-D/PRFI deviation indices	84.82	91.72	81.84	84.36	74.60	74.38	74.51
M4	M2 + pure Corvis DCR/stiffness parameters	84.99	90.95	82.78	84.60	74.29	74.09	75.16
M5	Full model without pure Corvis DCR/stiffness parameters	85.66	91.58	82.44	85.17	74.26	74.06	76.47
M6	Full model	**86.03**	**91.82**	**83.44**	**85.66**	**74.72**	**74.46**	**78.43**

DCR, dynamic corneal response. Values are presented as percentages. Stage-wise cross-validation metrics are reported as the mean performance across six candidate algorithms, including LR, DT, RF, SVM, XGBoost, and MLP. Stage 1: ICL vs Laser. Stage 2: SMILE vs SURFACE vs LASIK. Test ACC denotes the end-to-end four-class accuracy of the selected XGBoost-based Model B on the internal held-out test cohort under each feature set. Pure Corvis DCR/stiffness-related parameters refer to SSI, SP-A1, IR, and DA-Ratio. BAD-D and PRFI were analyzed separately as Pentacam-derived deviation/risk indices

Further addition of Pentacam-derived BAD-D/PRFI deviation indices increased the test accuracy to 74.51%, whereas adding pure Corvis ST dynamic corneal response and stiffness-related parameters to the tomography/pachymetry model increased the test accuracy to 75.16%. The full model without pure Corvis dynamic corneal response and stiffness-related parameters achieved a test accuracy of 76.47%. After adding SSI, SP-A1, IR, and DA-Ratio, the complete model achieved the highest test accuracy of 78.43%.

Therefore, compared with the full model already containing demographic/refractive variables, Pentacam tomographic and pachymetric parameters, Pentacam-derived risk indices, and pressure-/thickness-profile-/composite risk-related indices, the addition of pure Corvis ST dynamic corneal response and stiffness-related parameters showed a numerical improvement of 1.96 percentage points in end-to-end test accuracy. These findings suggest that pure Corvis ST-derived dynamic biomechanical variables may provide complementary predictive information, although the magnitude of this incremental contribution was modest.

To further quantify the incremental contribution of structured feature domains, paired patient-level clustered bootstrap comparisons were performed for predefined model pairs (Table 9). Adding Pentacam tomographic and pachymetric parameters to the demographic-refractive baseline model produced the largest improvement, increasing end-to-end accuracy by 26.14 percentage points (95% CI, 15.03 to 37.18), balanced accuracy by 29.36 percentage points (95% CI, 19.03 to 39.94), and macro-F1 by 27.27 percentage points (95% CI, 16.47 to 38.30).

**Table 9:** Paired patient-clustered bootstrap comparison of incremental feature contribution

Comparison	Increment tested	ΔAccuracy, pp (95% CI)	ΔBalanced accuracy, pp (95% CI)	ΔMacro-F1, pp (95% CI)	ΔWeighted-F1, pp (95% CI)	ΔMacro AUC, pp (95% CI)	Interpretation
M2 − M1	Pentacam tomography/pachymetry beyond demographic-refractive baseline	+26.14 (15.03 to 37.18)	+29.36 (19.03 to 39.94)	+27.27 (16.47 to 38.30)	+25.46 (14.05 to 36.76)	+19.69 (14.07 to 24.96)	Robust major improvement
M3 − M2	Pentacam BAD-D/PRFI deviation indices beyond tomography/pachymetry	+4.58 (−1.94 to 10.97)	+3.91 (−1.83 to 9.68)	+4.73 (−1.58 to 11.21)	+4.73 (−1.71 to 11.29)	+2.72 (0.69 to 5.08)	Numerical improvement; robust mainly for AUC
M4 − M2	Pure Corvis DCR/stiffness parameters beyond tomography/pachymetry	+5.23 (0.00 to 10.39)	+4.43 (−0.53 to 9.27)	+5.08 (−0.21 to 10.46)	+5.31 (0.03 to 10.54)	+1.37 (−0.32 to 3.18)	Modest improvement; classification gains should be interpreted cautiously
M6 − M5	Pure Corvis DCR/stiffness parameters beyond all non-pure-Corvis features	+1.96 (−4.77 to 9.09)	+1.82 (−4.64 to 8.30)	+1.60 (−5.34 to 8.62)	+2.13 (−4.76 to 9.28)	+0.84 (−0.65 to 2.33)	Strict incremental effect was small and not statistically robust

Values are presented as percentage-point differences. Differences were calculated as the performance of the expanded feature set minus that of the reference feature set in the end-to-end four-class task. Confidence intervals were estimated using paired patient-level clustered bootstrap resampling. In each bootstrap replicate, the same resampled patient set was used to evaluate both compared models, and all available eyes from selected patients were retained. DCR, dynamic corneal response. Pure Corvis DCR/stiffness parameters included SSI, SP-A1, IR, and DA-Ratio

The addition of Pentacam BAD-D/PRFI deviation indices beyond tomography/pachymetry produced a numerical improvement in classification metrics; however, the confidence intervals for accuracy, balanced accuracy, and macro-F1 crossed zero. The improvement in macro one-vs-rest AUC was 2.72 percentage points (95% CI, 0.69 to 5.08).

Adding pure Corvis ST dynamic corneal response and stiffness-related parameters to the tomography/pachymetry model improved end-to-end accuracy by 5.23 percentage points (95% CI, 0.00 to 10.39) and weighted-F1 by 5.31 percentage points (95% CI, 0.03 to 10.54), although the confidence intervals for balanced accuracy, macro-F1, and macro one-vs-rest AUC crossed or approached zero.

In the strictest comparison, the full model including pure Corvis ST dynamic corneal response and stiffness-related parameters was compared with the full model without these parameters. The additional gain was modest, with an accuracy improvement of 1.96 percentage points (95% CI, −4.77 to 9.09), balanced accuracy improvement of 1.82 percentage points (95% CI, −4.64 to 8.30), and macro-F1 improvement of 1.60 percentage points (95% CI, −5.34 to 8.62). These findings suggest that pure Corvis-derived dynamic biomechanical parameters may provide additional predictive information, but their strict incremental value beyond tomographic, pachymetric, and composite risk-related features was small and not statistically robust in the current dataset.

To further examine potential information overlap between Corvis ST dynamic biomechanical parameters and tomographic or risk-related variables, Spearman correlation analysis was performed between pure Corvis ST dynamic response/stiffness parameters and tomographic, pachymetric, deviation/risk, and composite risk-related variables. The correlation heatmap is shown in Figure S3, and the strongest correlations for each pure Corvis ST parameter are summarized in Table S9. Several moderate-to-strong correlations were observed. SP-A1 showed strong correlations with Dt (ρ = −0.731), TCT (ρ = 0.731), and CBI (ρ = −0.627). DA-Ratio was moderately to strongly correlated with Dt (ρ = 0.618), TCT (ρ = −0.617), and CBI (ρ = 0.563). IR showed moderate correlations with TCT (ρ = −0.521), Dt (ρ = 0.521), and Da (ρ = 0.498). In contrast, SSI showed weaker correlations, with the strongest correlation observed with bIOP (ρ = 0.349). These findings indicate partial information overlap between Corvis ST dynamic response/stiffness parameters and tomographic, pachymetric, or risk-related variables.

## Interpretability results

**Fig. 4:**
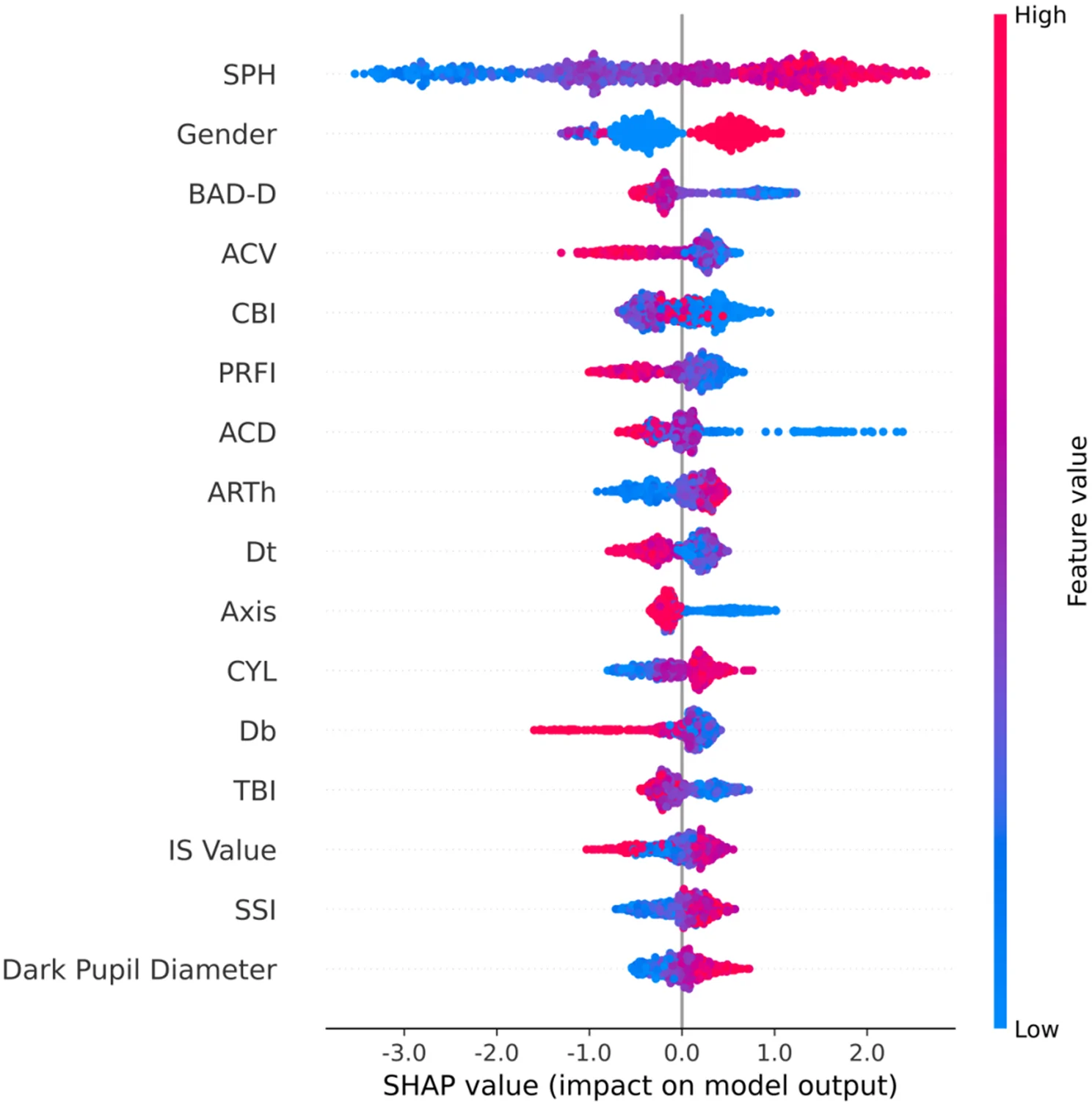
SHAP summary plot illustrating feature contributions in Stage 1 of model B. The laser procedure was labeled the positive class, and the ICL was labeled the negative class. The color gradient reflects the magnitude of each feature value, and SHAP values represent the direction and extent of their impact on model prediction

SHAP analysis was conducted to explore model behavior for both stages of Model B. In Stage 1, SPH was the highest-ranking feature for distinguishing ICL from corneal laser procedures, followed by tomographic, anterior-segment, and risk-related variables such as BAD-D, ACV, CBI, PRFI, ACD, ARTh, Dt, Db, TBI, IS Value, and SSI (Fig. 4).

**Fig. 5:**
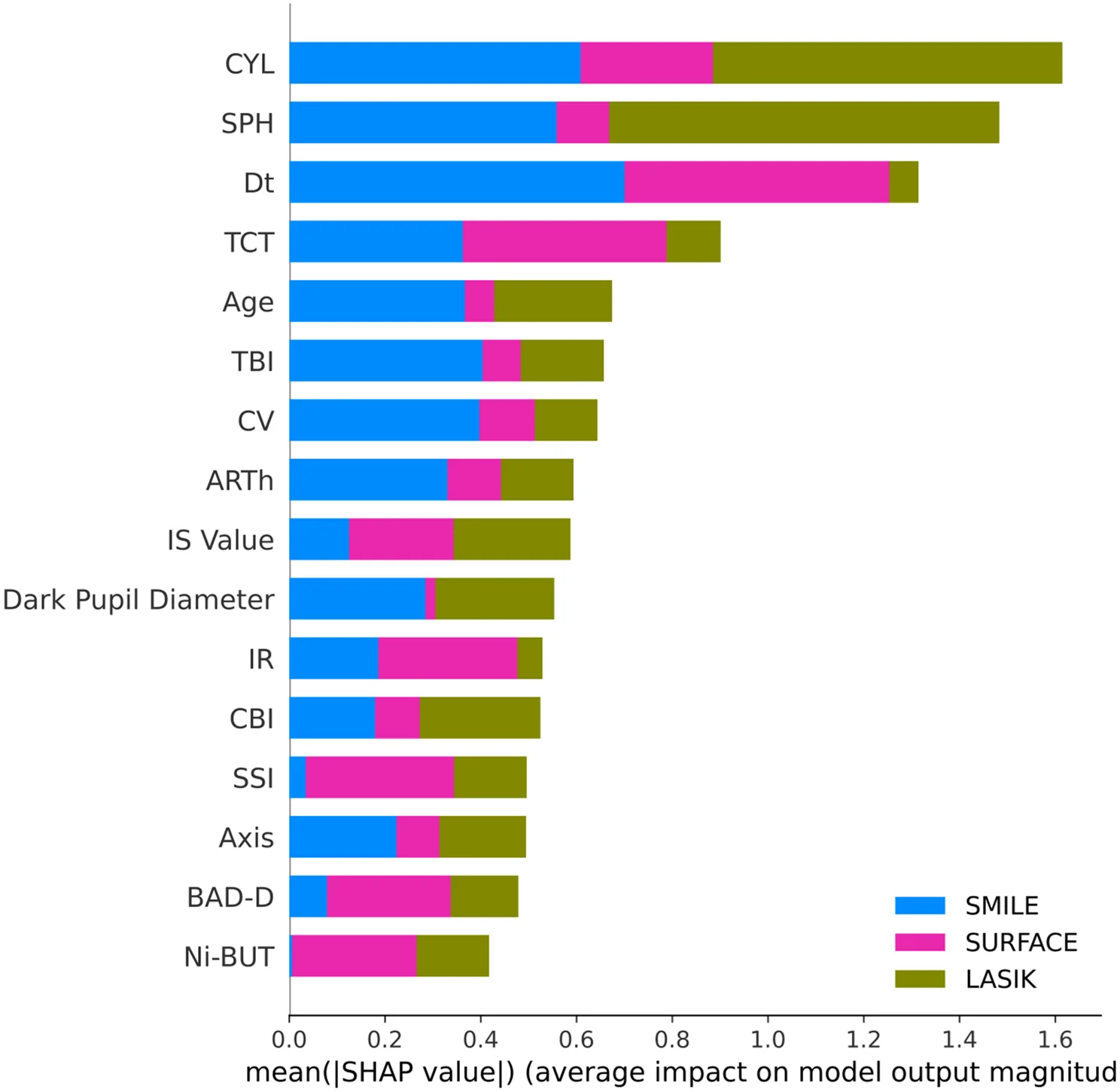
Global feature importance for the second-stage classifiers (SMILE, SURFACE, and LASIK). The mean absolute SHAP values quantify each feature’s contribution to model predictions

**Fig. 6:**
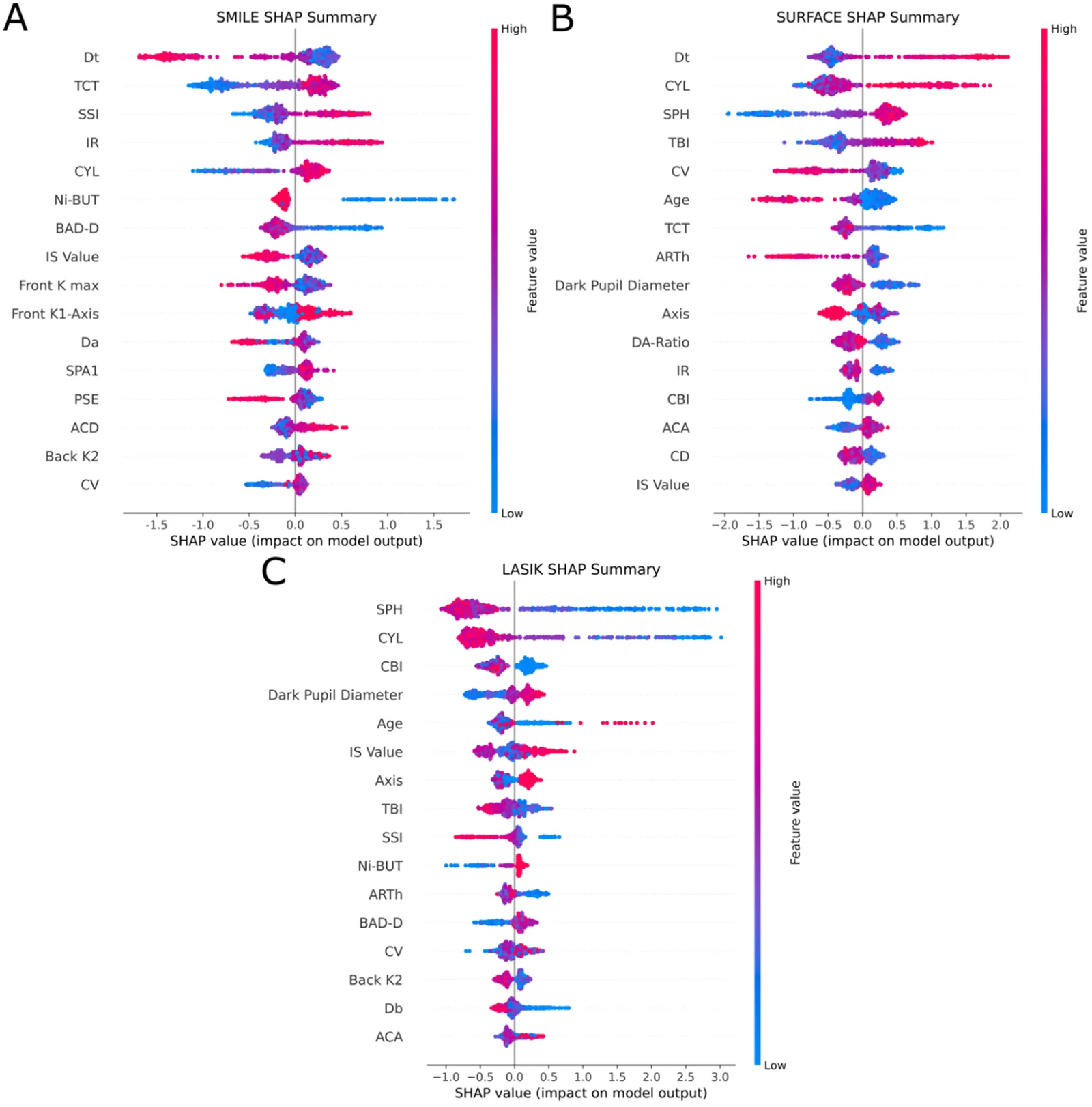
SHAP summary plots for the second-stage one-vs-rest classifiers distinguishing SMILE, SURFACE, and LASIK. The x-axis shows SHAP values (direction and magnitude of feature contribution), and the y-axis lists features. Each dot represents one sample; the color indicates the feature value (blue = low, red = high). Higher SHAP values increase the probability of the positive class, whereas lower values favor the negative classes. (**A**) SMILE vs others. (**B**) SURFACE vs others. (**C**) LASIK vs others

In Stage 2, the leading contributors to laser-subtype classification included CYL, SPH, Dt, TCT, Age, TBI, CV, ARTh, IS Value, Dark Pupil Diameter, IR, CBI, SSI, Axis, BAD-D, and Ni-BUT (Fig. 5). Class-specific one-vs-rest SHAP summary plots further showed distinct contribution patterns for SMILE, SURFACE, and LASIK (Fig. 6). These interpretability findings are described in detail in the Discussion.

## Discussion

This single-center retrospective study evaluated whether structured preoperative variables could reproduce clinician-selected refractive procedure patterns across ICL, SMILE, LASIK, and SURFACE. Rather than proposing a new machine learning algorithm or determining the objectively optimal procedure, the study focused on a clinically motivated task formulation, structured feature-domain comparison, and interpretable analysis of model behavior. The results showed that the two-stage Model B, which first separated ICL from corneal laser procedures and then classified laser procedures into SMILE, SURFACE, and LASIK, provided the most favorable balance between clinical interpretability and end-to-end performance. The additional stage in Model C did not improve final test performance, likely because downstream sample sizes became smaller and upstream errors could propagate through the hierarchical pathway. Therefore, the main value of this study lies in providing an internally validated analysis of single-center clinician-selected procedure patterns and in clarifying the relative contribution of different structured preoperative information domains, rather than in providing a deployable universal decision rule.

The expanded performance analysis also highlighted the importance of evaluating multiclass procedure-selection models beyond overall accuracy. Although Model B achieved an end-to-end accuracy of 78.43%, class-wise performance varied across surgical categories. SURFACE, the smallest category in the internal held-out test cohort, showed relatively lower precision despite acceptable recall, indicating that some non-SURFACE eyes were misclassified as SURFACE. Therefore, balanced accuracy, macro-F1 score, class-wise sensitivity, specificity, precision, one-vs-rest AUC, and confusion matrices are necessary for interpreting model performance in imbalanced refractive surgery datasets. Calibration analysis further suggested that predicted probabilities were not perfectly aligned with observed frequencies, particularly in smaller categories. Thus, model probabilities should not be directly interpreted as calibrated clinical risk estimates without further validation or recalibration.

A central analytical consideration in this study was the distinction between pure Corvis ST-derived dynamic corneal response and stiffness-related parameters and other tomographic, pachymetric, or composite risk-related indices. BAD-D and PRFI primarily reflect Pentacam-derived tomographic or morphological deviation information; ARTh is closely related to corneal thickness distribution; and TBI integrates both tomographic and biomechanical information. Therefore, grouping all of these variables as “biomechanical features” would overestimate the contribution of pure biomechanical information. In the structured feature-domain analysis, most predictive gain came from Pentacam tomographic and pachymetric parameters. Pure Corvis ST parameters, including SSI, SP-A1, IR, and DA-Ratio, provided additional numerical improvement, but their strict incremental contribution beyond the full non-pure-Corvis feature set was modest and not statistically robust. The supplementary correlation analysis further showed that SP-A1 and DA-Ratio were moderately to strongly correlated with TCT, Dt, and CBI, while IR showed moderate correlations with TCT, Dt, and Da. These findings suggest that Corvis ST-derived dynamic response/stiffness parameters are not completely independent from tomographic, pachymetric, or risk-related variables. Such information overlap may partly explain the limited strict incremental gain of pure Corvis ST parameters and supports interpreting them as complementary and partially overlapping predictors.

The interpretation of elevated TBI values also requires caution. TBI is a composite tomographic-biomechanical index and should not be interpreted as an isolated diagnosis of clinical keratoconus or as an absolute surgical contraindication. The commonly cited cutoff of 0.29 was optimized for detecting very asymmetric ectasia fellow eyes with normal topography, whereas higher cutoffs have been reported for clinical ectasia; thresholds may also vary according to disease definition, software version, and population. In this cohort, elevated TBI values were therefore reviewed in the context of comprehensive tomographic, pachymetric, biomechanical, and clinical findings. The relatively higher TBI values in the ICL and SURFACE groups may reflect risk-stratified clinical allocation, in which eyes with greater tomographic-biomechanical susceptibility were more often directed toward ICL or selected surface ablation rather than flap-based procedures. However, although no postoperative ectasia or keratoconus progression was documented in available records, systematic long-term outcome follow-up was not incorporated. These findings should therefore not be interpreted as evidence that elevated-TBI eyes are generally suitable for corneal refractive surgery or that the selected procedures are definitively safe.

SHAP analysis was used to explore model behavior rather than to infer causal clinical importance or objective surgical decision rules. In Stage 1, refractive status remained the dominant factor separating ICL from corneal laser procedures, with SPH ranking as the most influential feature. Lower SPH values contributed more strongly toward ICL prediction, consistent with the tendency to avoid excessive corneal tissue removal in eyes with high myopia or limited corneal reserve [29–31]. Several tomographic, anterior-segment, and risk-related variables, including BAD-D, ACV, CBI, PRFI, ACD, ARTh, Dt, Db, TBI, IS Value, and SSI, also contributed to Stage 1 predictions. These findings suggest that the model reproduced the ICL-versus-laser boundary using refractive status together with multiparametric corneal and anterior-segment information, rather than relying on any single risk index. Although Gender appeared among the high-ranking Stage 1 features, this should not be interpreted as a causal clinical determinant of procedure selection. It may reflect cohort-specific demographic imbalance or local practice patterns and requires validation in larger external datasets.

In Stage 2, laser-subtype prediction was shaped mainly by refractive status, tomographic and pachymetric deviation, corneal thickness reserve, and composite risk-related information. CYL, SPH, Dt, TCT, Age, TBI, CV, ARTh, IS Value, Dark Pupil Diameter, IR, CBI, SSI, Axis, BAD-D, and Ni-BUT were among the leading contributors. Class-specific SHAP patterns suggested that SMILE predictions were associated with relatively favorable corneal thickness reserve and dynamic response profiles, whereas SURFACE predictions were more influenced by higher TBI or tomographic deviation-related signals together with lower thickness-profile reserve. LASIK predictions were driven mainly by refractive parameters and were reduced by higher composite risk-related signals such as CBI and TBI. These patterns are broadly consistent with known differences in biomechanical effects among corneal refractive procedures [23, 24, 26, 32–34]. Nevertheless, because cap thickness, flap thickness, residual stromal bed, ablation depth, lenticule thickness, and percent tissue altered were not included as model inputs, and because several structured variables were correlated, SHAP findings should be interpreted as exploratory explanations of single-center model behavior rather than evidence of procedure-specific superiority or isolated causal importance.

Several limitations should be acknowledged. First, the outcome label represented the procedure actually selected and performed by clinicians rather than an independently adjudicated optimal procedure or a long-term postoperative safety endpoint. Therefore, the model learned historical procedure-selection patterns under local clinical protocols, surgeon judgment, and patient preference. This design reflects real-world clinical practice but cannot determine whether an alternative procedure would have achieved better visual, refractive, biomechanical, safety, or patient-reported outcomes.

Second, this was a single-center study, and refractive procedure selection is inherently dependent on institution-specific resources and workflows. Available surgical platforms, laser systems, treatment nomograms, cap/flap or lenticule-planning settings, tissue-removal strategies, ICL sizing protocols, surgeon experience, treatment cost, and patient preference may all influence final procedure choice. Although procedures were selected and performed by three experienced refractive surgeons following a shared institutional workflow, individual surgeon identity, surgeon-specific preference, detailed patient preference, cost considerations, and platform-specific decision factors were not available as structured model inputs. These unmeasured non-anatomical and non-biomechanical factors are particularly relevant for patients who are technically suitable for more than one procedure. The model may therefore partly capture institutional practice patterns and preference-sensitive decision-making rather than purely objective clinical suitability. Accordingly, the model should not be interpreted as an absolute or universally applicable procedure-selection rule, and multicenter patient-level external validation with more complete decision-context variables is required before broader clinical application.

Third, although all data partitions were performed at the patient level and additional patient-level clustered bootstrap and one-eye-per-patient sensitivity analyses were conducted, the effective independent sample size remained limited, and some confidence intervals were relatively wide. Procedure categories were also imbalanced, and the model was restricted to structured preoperative variables without incorporating raw imaging data, Corvis deformation waveforms, detailed patient preference variables, or postoperative outcome endpoints. TCT was included as the primary corneal thickness-related model input, whereas CCT, residual stromal bed, ablation depth, lenticule thickness, and percent tissue altered were not incorporated. These parameters are clinically important for corneal laser procedure planning, but they depend on the selected or simulated treatment profile and may represent post-decision information in a model designed to predict clinician-selected procedures. Their exclusion reduced the risk of label leakage but limited direct representation of tissue-removal safety margins. Finally, decision-curve analysis was not performed because the endpoint was clinician-selected procedure rather than postoperative outcome-defined benefit or adverse event. Outcome-based decision-curve analysis should be considered in future studies incorporating visual, refractive, biomechanical, and safety endpoints.

Collectively, these findings suggest that structured preoperative variables can partially reproduce single-center clinician-selected refractive procedure patterns. Corvis ST-derived dynamic biomechanical parameters may provide complementary predictive information, but the present study does not establish optimal surgical selection, clinical outcome benefit, or broad external generalizability.

## Conclusion

This single-center retrospective study showed that a clinically oriented and interpretable machine learning model could partially reproduce clinician-selected refractive procedure patterns across ICL, SMILE, LASIK, and SURFACE using structured preoperative variables. Corvis ST-derived dynamic biomechanical parameters suggested modest complementary predictive information beyond conventional refractive, tomographic, pachymetric, and risk-related features, but they should be interpreted as partially overlapping rather than fully independent predictors. However, the outcome label represented the procedure actually selected by clinicians rather than an independently verified optimal or safest procedure. Therefore, the findings should be interpreted as internally validated evidence of single-center procedure-selection pattern prediction and as an analytical baseline for future research, not as definitive clinical guidance. Multicenter patient-level external validation and outcome-based studies incorporating postoperative visual, refractive, biomechanical, safety, and patient-reported endpoints are required before any clinical decision-support application.

## Electronic supplementary material

Below is the link to the electronic supplementary material.

Supplementary Material 1

Supplementary Material 2

## Data Availability

The datasets used and/or analysed during the current study are available from the corresponding author on reasonable request.

## Abbreviations

ICL: Implantable Collamer Lens
LASIK: Laser-assisted in situ keratomileusis
SHAP: Shapley additive explanations
LASEK: Laser-assisted subepithelial keratectomy
PRK: Photorefractive Keratectomy
Trans-PRK: Transepithelial PRK
SURFACE: Surface ablation (including PRK, Trans-PRK, LASEK)
SMILE: Small Incision Lenticule Extraction
DCR: Dynamic corneal response
DT: Decision Tree
RF: Random Forest
LR: Logistic Regression
SVM: Support Vector Machine
MLP: Multilayer Perceptron
XGBoost: eXtreme Gradient Boosting
SMOTE: Synthetic Minority Oversampling Technique
AUC: Area Under the Curve
SRI: Surface Regularity Index
SAI: Surface Asymmetry Index
CSI: Corneal Symmetry Index
RSB: Residual Stromal Bed

## References

[1] Song S, Wen D, Yin Y, Qian F, Xu H, Xia X. Correction of presbyopia. Zhong Nan Da Xue Xue Bao Yi Xue Ban. 2022;47(10):1454–60. 10.11817/j.issn.1672-7347.2022.220201. 36411697 10.11817/j.issn.1672-7347.2022.220201 PMC10930369

[2] Wang Q, Fan L, Zhou Q. The best choice for low and moderate myopia patients incapable for corneal refractive surgery: implantation of a posterior chamber phakic intraocular lens. Int Ophthalmol. 2022;43(2):575–81. 10.1007/s10792-022-02459-3. 35984557 10.1007/s10792-022-02459-3 PMC9971141

[3] Swaminathan U, Daigavane S. Comparative analysis of visual outcomes and complications in intraocular collamer lens, small-incision lenticule extraction, and laser-assisted in situ keratomileusis surgeries: a comprehensive review. Cureus. 2024;16(4). 10.7759/cureus.58718. 10.7759/cureus.58718 PMC11110473 38779265

[4] Chen Q, Yan H, Chen L, Wang L, Zheng Q, Ren Y. Adverse events associated with implantable collamer lens: insights from the FDA MAUDE database. Front Med. 2025;12:1613060. 10.3389/fmed.2025.1613060. 10.3389/fmed.2025.1613060 PMC12319018 40761863

[5] Di Y, Li Y, Luo Y. Prediction of implantable collamer lens vault based on preoperative biometric factors and lens parameters. J Refract Surg. 2023;39(5):332–39. 10.3928/1081597X-20230207-03. 37162400 10.3928/1081597X-20230207-03

[6] Yoo TK, Ryu IH, Lee G, Kim Y, Kim JK, Lee IS, et al. Adopting machine learning to automatically identify candidate patients for corneal refractive surgery. NPJ Digit Med. 2019;2(1):59. 10.1038/s41746-019-0135-8. 31304405 10.1038/s41746-019-0135-8 PMC6586803

[7] Ting DSW, Pasquale LR, Peng L, Campbell JP, Lee AY, Raman R, et al. Artificial intelligence and deep learning in ophthalmology. Br J Ophthalmol. 2019;103(2):167–75. 10.1136/bjophthalmol-2018-313173. 30361278 10.1136/bjophthalmol-2018-313173 PMC6362807

[8] Yoo TK, Ryu IH, Choi H, Kim JK, Lee IS, Kim JS, et al. Explainable machine learning approach as a tool to understand factors used to select the refractive surgery technique on the expert level. Trans Vis Sci Tech. 2020;9(2):8–8. 10.1167/tvst.9.2.8. 10.1167/tvst.9.2.8 PMC7346876 32704414

[9] Cui T, Wang Y, Ji S, Li Y, Hao W, Zou H, et al. Applying machine learning techniques in nomogram prediction and analysis for SMILE treatment. Am J Ophthalmol. 2020;210:71–77. 10.1016/j.ajo.2019.10.015. 10.1016/j.ajo.2019.10.015 31647929

[10] Wang K, Xu C, Li G, Zhang Y, Zheng Y, Sun C. Combining convolutional neural networks and self-attention for fundus diseases identification. Sci Rep. 2023;13(1):76. 10.1038/s41598-022-27358-6. 36593268 10.1038/s41598-022-27358-6 PMC9807560

[11] Li J, Dai Y, Mu Z, Wang Z, Meng J, Meng T, et al. Choice of refractive surgery types for myopia assisted by machine learning based on doctors’ surgical selection data. BMC Med Inf Decis Mak. 2024;24(1):41. 10.1186/s12911-024-02451-0. 10.1186/s12911-024-02451-0 PMC10854042 38331788

[12] Li L, Xiang Y, Chen X, Lin D, Zhao L, Xiao J, et al. Machine learning model for predicting corneal stiffness and identifying keratoconus based on ocular structures. Intell Med. 2025;5(1):66–72. 10.1016/j.imed.2024.09.006.

[13] Choi JY, Choi H, Cho Y, Yoo TK. Artificial intelligence and refractive surgeries including laser vision correction and phakic IOL implantation—a narrative review. Ann Eye Sci. 2025;10:7–7. 10.21037/aes-24-40.

[14] Ambrósio, Jr JR, Correia FF, Lopes B, Salomão MQ, Luz A, Dawson DG, et al. Corneal biomechanics in ectatic diseases: refractive surgery implications. Open Ophthalmol J. 2017;11(1):176. 10.2174/1874364101711010176. 28932334 10.2174/1874364101711010176 PMC5585467

[15] Steinberg J, Siebert M, Katz T, Frings A, Mehlan J, Druchkiv V, et al. Tomographic and biomechanical Scheimpflug imaging for keratoconus characterization: a validation of current indices. J Refract Surg. 2018;34(12):840–47. 10.3928/1081597X-20181012-01. 30540367 10.3928/1081597X-20181012-01

[16] Moshirfar M, Motlagh MN, Murri MS, Momeni-Moghaddam H, Ronquillo YC, Hoopes PC. Advances in biomechanical parameters for screening of refractive surgery candidates: a review of the literature, part III. Med Hypothesis Discov Innov Ophthalmol. 2019;8(3):219. 31598522 PMC6778467

[17] Yang K, Xu L, Fan Q, Zhao D, Ren S. Repeatability and comparison of new Corvis ST parameters in normal and keratoconus eyes. Sci Rep. 2019;9(1):15379. 10.1038/s41598-019-51502-4. 31653884 10.1038/s41598-019-51502-4 PMC6814725

[18] Liu Y, Zhang Y, Chen Y. Application of a scheimpflug-based biomechanical analyser and tomography in the early detection of subclinical keratoconus in Chinese patients. BMC Ophthalmol. 2021;21(1):339. 10.1186/s12886-021-02102-2. 34544392 10.1186/s12886-021-02102-2 PMC8454178

[19] Esporcatte LPG, Salomão MQ, Junior NS, Machado AP, Ferreira É, Loureiro T, et al. Corneal biomechanics for corneal ectasia: update. Saudi J Ophthalmol. 2022;36(1):17–24. 10.4103/sjopt.sjopt_192_21. 35971484 10.4103/sjopt.sjopt_192_21 PMC9375464

[20] Borderie V, Beauruel J, Cuyaubère R, Georgeon C, Memmi B, Sandali O. Comprehensive assessment of Corvis ST biomechanical indices in normal and keratoconus corneas with reference to corneal enantiomorphism. J Clin Med. 2023;12(2):690. 10.3390/jcm12020690. 36675618 10.3390/jcm12020690 PMC9863401

[21] Damgaard IB, Reffat M, Hjortdal J. Review of corneal biomechanical properties following LASIK and SMILE for myopia and myopic astigmatism. Open Ophthalmol J. 2018;12(1):164. 10.2174/1874364101812010164. 30123381 10.2174/1874364101812010164 PMC6062908

[22] Guo H, Hosseini-Moghaddam SM, Hodge W. Corneal biomechanical properties after SMILE versus FLEX, LASIK, LASEK, or PRK: a systematic review and meta-analysis. BMC Ophthalmol. 2019;19(1):167. 10.1186/s12886-019-1165-3. 31370817 10.1186/s12886-019-1165-3 PMC6676534

[23] Xin Y, Lopes BT, Wang J, Wu J, Zhu M, Jiang M, et al. Biomechanical effects of tPRK, FS-LASIK, and SMILE on the cornea. Front Bioeng Biotechnol. 2022;10:834270. 10.3389/fbioe.2022.834270. 35433653 10.3389/fbioe.2022.834270 PMC9009506

[24] Hashemi H, Roberts CJ, Elsheikh A, Mehravaran S, Panahi P, Asgari S. Corneal biomechanics after SMILE, femtosecond-assisted LASIK, and photorefractive keratectomy: a matched comparison study. Trans Vis Sci Tech. 2023;12(3):12–12. 10.1167/tvst.12.3.12. 10.1167/tvst.12.3.12 PMC10029763 36928130

[25] Qu Z, Li X, Yuan Y, Wang P, Li Y, Lin S, et al. In vivo corneal biomechanical response to three different laser corneal refractive surgeries. J Refract Surg. 2024;40(5):e344–52. 10.3928/1081597X-20240322-01. 10.3928/1081597X-20240322-01 38717086

[26] Joshi S, Bari A, Shakkarwal C, Agarwal T, Dada T, Sinha R, et al. The visual outcomes and corneal biomechanical properties after PRK and SMILE in low to moderate myopia. Indian J Ophthalmol. 2025;73(1):128–33. 10.4103/IJO.IJO_1250_24. 39446853 10.4103/IJO.IJO_1250_24 PMC11831955

[27] Shortt AJ, Allan BD, Evans JR. Laser-assisted in-situ keratomileusis (LASIK) versus photorefractive keratectomy (PRK) for myopia. Cochrane Database Syst Rev. 2013;2013(1):1. 10.1002/14651858.CD005135.pub3. 10.1002/14651858.CD005135.pub3 PMC11848121 23440799

[28] Jacobs DS, Lee JK, Shen TT, Afshari NA, Bishop RJ, Keenan JD, et al. Refractive surgery preferred practice pattern®. Ophthalmology. 2023;130(3):PP61–P135. 10.1016/j.ophtha.2022.10.032. 10.1016/j.ophtha.2022.10.032 36543604

[29] Wang X, Zhou X. Update on treating high myopia with implantable collamer lenses. Asia Pac J Ophthalmol. 2016;5(6):445–49. 10.1097/APO.0000000000000235. 10.1097/APO.0000000000000235 27898450

[30] Kamiya K, Takahashi M, Takahashi N, Shoji N, Shimizu K. Monovision by implantation of posterior chamber phakic intraocular lens with a central hole (hole ICL) for early presbyopia. Sci Rep. 2017;7(1):11302. 10.1038/s41598-017-11539-9. 28900204 10.1038/s41598-017-11539-9 PMC5595884

[31] Li F, Ma Y, Qi W, Pazo EE, Yang R, Zhao S. Characteristics of biological parameters and implantable collamer lens (ICL) size selection in moderate, high, and super-high myopia eyes. BMC Ophthalmol. 2025;25(1):103. 10.1186/s12886-025-03934-y. 40033255 10.1186/s12886-025-03934-y PMC11874806

[32] Reinstein DZ, Archer TJ, Randleman JB. Mathematical model to compare the relative tensile strength of the cornea after PRK, LASIK, and small incision lenticule extraction. J Refract Surg. 2013;29(7):454–60. 10.3928/1081597X-20130617-03. 23820227 10.3928/1081597X-20130617-03

[33] Roy AS, Dupps WJ Jr, Roberts CJ. Comparison of biomechanical effects of small-incision lenticule extraction and laser in situ keratomileusis: finite-element analysis. J Cataract Refract Surg. 2014;40(6):971–80. 10.1016/j.jcrs.2013.08.065. 10.1016/j.jcrs.2013.08.065 PMC6030688 24857440

[34] Gao W, Zhao X, Wang Y. Change in the corneal material mechanical property for small incision lenticule extraction surgery. Front Bioeng Biotechnol. 2023;11:1034961. 10.3389/fbioe.2023.1034961. 36890912 10.3389/fbioe.2023.1034961 PMC9986312

