# Supplementary material for: Incremental contribution of Corvis ST dynamic biomechanical parameters to interpretable machine learning prediction of clinician-selected refractive procedures: a retrospective observational study
Source: BMC Ophthalmol. 2026 Aug 20;26:516. doi: 10.1186/s12886-026-05208-7 (PMC13501657; doi:10.1186/s12886-026-05208-7)
Supplement: Supplementary file 1 [file 12886_2026_5208_MOESM1_ESM.pdf]

Algorithm 1 Patient-level model development and evaluation workflow

Require: Eye-level dataset $\mathcal{D} = \{(\mathbf{x}_i, y_i, p_i)\}_{i=1}^{n}$, where $\mathbf{x}_i$ denotes structured preoperative variables, y_i denotes the clinician-selected procedure label, and p_i denotes the patient identifier
Require: Candidate model architectures $\mathcal{M} = \{\text{Model A}, \text{Model B}, \text{Model C}\}$
Require: Candidate algorithms $\mathcal{A} = \{\text{LR}, \text{DT}, \text{RF}, \text{SVM}, \text{XGBoost}, \text{MLP}\}$
Require: Predefined hyperparameter grids $\mathcal{G}$ for Stage 1 and Stage 2 classifiers
Require: Global random seed $s = 2026$; SMOTE setting $\text{SMOTE}(\text{random\_state} = 7)$

Split $\mathcal{D}$ into training cohort $\mathcal{D}_{\text{train}}$ and internal held-out test cohort $\mathcal{D}_{\text{test}}$ at the patient level using an 8:2 ratio
Reserve $\mathcal{D}_{\text{test}}$ and exclude it from preprocessing, feature selection, hyperparameter tuning, algorithm selection, and architecture selection
Define patient-grouped fivefold cross-validation folds $\{\mathcal{V}_k\}_{k=1}^{5}$ within $\mathcal{D}_{\text{train}}$ using patient identifiers as grouping labels
for each candidate architecture $m \in \mathcal{M}$ **do**
  for each candidate algorithm $a \in \mathcal{A}$ **do**
    for each hyperparameter setting $g \in \mathcal{G}_a$ **do**
      for each fold $k = 1, \ldots, 5$ **do**
        Define fold-specific training subset $\mathcal{D}_{\text{tr}}^{(k)}$ and validation subset $\mathcal{D}_{\text{val}}^{(k)}$ according to patient-level grouping
        Fit preprocessing pipeline $\Pi^{(k)}$ only on $\mathcal{D}_{\text{tr}}^{(k)}$
        ▷ $\Pi^{(k)}$ includes Z-score standardization, Pearson correlation filtering, and low-variance feature filtering
        Transform $\mathcal{D}_{\text{tr}}^{(k)}$ and $\mathcal{D}_{\text{val}}^{(k)}$ using $\Pi^{(k)}$
        Apply SMOTE oversampling only to the transformed training subset $\Pi^{(k)}(\mathcal{D}_{\text{tr}}^{(k)})$
        Train classifier $f_{m,a,g}^{(k)}$ on the preprocessed and oversampled training subset
        Evaluate $f_{m,a,g}^{(k)}$ on the preprocessed validation subset $\Pi^{(k)}(\mathcal{D}_{\text{val}}^{(k)})$ without oversampling
      end for
      Aggregate validation performance across the five patient-grouped folds
    end for
  end for
end for
Select the final model using only cross-validation performance within $\mathcal{D}_{\text{train}}$
Retrain the selected final model f^* on the full training cohort $\mathcal{D}_{\text{train}}$ using the same strategy
Apply the fitted pipeline and f^* once to the untouched internal held-out test cohort $\mathcal{D}_{\text{test}}$
Calculate Stage 1, Stage 2, and final end-to-end performance metrics on $\mathcal{D}_{\text{test}}$
Estimate 95% confidence intervals using patient-level clustered bootstrap resampling
Conduct one-eye-per-patient sensitivity analysis by randomly selecting one eye from each patient in $\mathcal{D}_{\text{test}}$ and repeating the procedure 1,000 times
Perform calibration analysis on $\mathcal{D}_{\text{test}}$ without fitting any additional model
for each predefined structured feature set $\mathcal{F}_r$ **do**
  Repeat the same grouping, preprocessing, SMOTE strategy, candidate-algorithm comparison, and hyperparameter search procedure using variables in $\mathcal{F}_r$
  Evaluate the selected model for $\mathcal{F}_r$ on $\mathcal{D}_{\text{test}}$
end for
Perform paired patient-level clustered bootstrap comparisons between predefined feature-set models to quantify incremental feature-domain contribution
return Selected model, internal held-out test performance, uncertainty estimates, sensitivity results, calibration metrics, and ablation comparison results
