# Supplementary material for: Incremental contribution of Corvis ST dynamic biomechanical parameters to interpretable machine learning prediction of clinician-selected refractive procedures: a retrospective observational study
Source: BMC Ophthalmol. 2026 Aug 20;26:516. doi: 10.1186/s12886-026-05208-7 (PMC13501657; doi:10.1186/s12886-026-05208-7)
Supplement: Supplementary file 2 [file 12886_2026_5208_MOESM2_ESM.docx]

**Supplemental material**

Table S1. Structured preoperative variables included in the model, data sources, and feature-domain codes.

Table S1A. Feature-domain codes and definitions.

| **Domain code** | **Feature domain** |
| --- | --- |
| D1 | Demographic, refractive, visual, and ocular-surface baseline characteristics |
| D2 | Pentacam tomographic, keratometric, pachymetric, and anterior-segment parameters |
| D3 | Pentacam-derived ectasia-risk and deviation indices |
| D4 | Corvis ST dynamic corneal response and stiffness-related parameters |
| D5 | Pressure-, thickness-profile-, and composite risk-related indices |

Table S1B. Structured preoperative variables included in the model.

| **Index** | **Abbreviation** | **Full name** | **Source** | **Domain code** |
| --- | --- | --- | --- | --- |
| 1 | - | Gender | Clinical record | D1 |
| 2 | - | Age | Clinical record | D1 |
| 3 | NiBUT | Non-invasive Break-Up Time | Ocular surface examination | D1 |
| 4 | SPH | Spherical Refraction | Refraction examination | D1 |
| 5 | CYL | Cylindrical Power | Refraction examination | D1 |
| 6 | Axis | Astigmatism Axis | Refraction examination | D1 |
| 7 | SE | Spherical Equivalent | Refraction examination | D1 |
| 8 | UDVA | Uncorrected Distance Visual Acuity | Visual acuity examination | D1 |
| 9 | CDVA | Corrected Distance Visual Acuity | Visual acuity examination | D1 |
| 10 | - | Dark Pupil Diameter | clinical preoperative examination | D1 |
| 11 | Front K1 | Anterior Corneal Flat Keratometry | Pentacam HR | D2 |
| 12 | Front K2 | Anterior Corneal Steep Keratometry | Pentacam HR | D2 |
| 13 | Front K mean | Mean Anterior Corneal Keratometry | Pentacam HR | D2 |
| 14 | Front K1 Axis | Axis of Anterior Corneal Flat Keratometry | Pentacam HR | D2 |
| 15 | Back K1 | Posterior Corneal Flat Keratometry | Pentacam HR | D2 |
| 16 | Back K2 | Posterior Corneal Steep Keratometry | Pentacam HR | D2 |
| 17 | Back K mean | Mean Posterior Corneal Keratometry | Pentacam HR | D2 |
| 18 | Back Axis K1 | Axis of Posterior Corneal Flat Keratometry | Pentacam HR | D2 |
| 19 | TCT | Thinnest Corneal Thickness | Pentacam HR | D2 |
| 20 | CV | Corneal Volume | Pentacam HR | D2 |
| 21 | ACV | Anterior Chamber Volume | Pentacam HR | D2 |
| 22 | ACA | Anterior Chamber Angle | Pentacam HR | D2 |
| 23 | ACD | Anterior Chamber Depth | Pentacam HR | D2 |
| 24 | Front K max | Maximum Anterior Corneal Keratometry | Pentacam HR | D2 |
| 25 | Q Value | Corneal Asphericity | Pentacam HR | D2 |
| 26 | - | Anterior Surface Elevation at TCT | Pentacam HR | D2 |
| 27 | - | Posterior Surface Elevation at TCT | Pentacam HR | D2 |
| 28 | Pupil Center X | Horizontal Coordinate of Pupil Center | Pentacam HR | D2 |
| 29 | Pupil Center Y | Vertical Coordinate of Pupil Center | Pentacam HR | D2 |
| 30 | Angle Kappa | Angle Kappa | Pentacam HR | D2 |
| 31 | CD | Corneal Diameter | Pentacam HR | D2 |
| 32 | IS Value | Inferior–Superior Value | Pentacam HR | D2 |
| 33 | - | Average Pachymetric Progression Rate | Pentacam HR | D2 |
| 34 | Df | Standard Deviation of Front Surface Height | Pentacam HR | D3 |
| 35 | Db | Standard Deviation of Back Surface Height | Pentacam HR | D3 |
| 36 | Dp | Standard Deviation of Pachymetric Progression | Pentacam HR | D3 |
| 37 | Dt | Standard Deviation of Thinnest Point Thickness | Pentacam HR | D3 |
| 38 | Da | Standard Deviation of Ambrosio Relational Thickness | Pentacam HR | D3 |
| 39 | BAD-D | Belin–Ambrosio Enhanced Ectasia Display Deviation Index | Pentacam HR | D3 |
| 40 | PRFI | Pentacam Random Forest Index | Pentacam HR | D3 |
| 41 | SSI | Stress–Strain Index | Corvis ST | D4 |
| 42 | SP-A1 | Stiffness Parameter at First Applanation | Corvis ST | D4 |
| 43 | IR | Integrated Radius | Corvis ST | D4 |
| 44 | DA-Ratio | Deformation Amplitude Ratio 2 mm | Corvis ST | D4 |
| 45 | bIOP | Biomechanically Corrected Intraocular Pressure | Corvis ST | D5 |
| 46 | ARTh | Ambrosio Relational Thickness horizontal | Pentacam HR / Corvis ST report | D5 |
| 47 | CBI | Corvis Biomechanical Index | Corvis ST | D5 |
| 48 | TBI | Tomographic and Biomechanical Index | Pentacam HR / Corvis ST report | D5 |

Table S2. Kruskal-Wallis test results comparing baseline and preoperative variables among clinician-selected procedure groups.

| **Feature Name** | **P Value** | **Feature Name** | **P Value** |
| --- | --- | --- | --- |
| Gender | 0.5973 | Q Value | 0.7609 |
| Age | **<0.01** | Anterior Surface Elevation at the Corneal Thinnest Point | 0.5413 |
| NiBUT (Non-invasive Break Up Time) | **<0.01** | Posterior Surface Elevation at the Corneal Thinnest Point | 0.5779 |
| SPH (Spherical Refraction) | **<0.01** | Df (Std of Front Surface Height) | 0.1314 |
| CYL (Cylindrical Power) | **<0.01** | Db (Std of Back Surface Height) | **<0.01** |
| Axis | **<0.01** | Dp (Std of Pachymetric Progression) | **<0.01** |
| SE (Spherical Equivalent) | **<0.01** | Dt (Std of Thinnest Point Thickness) | **<0.01** |
| UDVA (Uncorrected Distance Vision Acuity) | **<0.01** | Da (Std of Ambrosio Relative Thickness) | **<0.01** |
| CDVA (Corrected Distance Vision Acuity) | **<0.01** | Pupil Center X | 0.8540 |
| Dark Pupil Diameter | 0.2550 | Pupil Center Y | 0.3385 |
| Front K1 | 0.0539 | Angle Kappa | 0.6808 |
| Front K2 | **<0.01** | CD (Corneal Diameter) | 0.2571 |
| Front K mean | 0.0344 | IS Value (Inferior-Superior Value) | 0.4444 |
| Front K1 Axis | 0.4520 | Average Pachymetric Progression Rate | **<0.01** |
| Back K1 | 0.0995 | bIOP (Biomechanically corrected Intra-Ocular Pressure) | 0.4303 |
| Back K2 | 0.0217 | PRFI (Pentacam Random Forest Index) | **<0.01** |
| Back K mean | 0.1212 | SSI (Stress-Strain Index) | **<0.01** |
| Back Axis K1 | 0.2878 | SP-A1 (Stiffness Parameter at First Applanation) | **<0.01** |
| TCT (Thinnest Corneal Thickness) | **<0.01** | IR (Integrated Radius) | **<0.01** |
| CV (Corneal Volume) | **<0.01** | ARTh (Ambrosio Relational Thickness horizontal) | **<0.01** |
| ACV (Anterior Chamber Volume) | 0.6991 | DA-Ratio (Deformation Amplitude Ratio 2 mm) | **<0.01** |
| ACA (Anterior Chamber Angle) | 0.1543 | CBI (Corvis Biomechanical Index) | **<0.01** |
| ACD (Anterior Chamber Depth) | 0.4535 | BAD-D (Belin/Ambrosio Display-Deviation) | **<0.01** |
| Front K max | **<0.01** | TBI (Tomographic and Biomechanical Index) | **<0.01** |

Table S3. Patient-grouped cross-validation performance of candidate machine learning algorithms across model architectures.

| **Model** | **Stage** | **Metric** | **LR** | **DT** | **RF** | **SVM** | **MLP** | **XGBoost** |
| --- | --- | --- | --- | --- | --- | --- | --- | --- |
| Model A | Four-class | ACC | 60.41 ± 4.35 | 52.57 ± 4.65 | **78.38 ± 4.78** | 58.24 ± 4.28 | 69.05 ± 2.15 | 77.70 ± 3.34 |
| Model A | Four-class | Macro Recall | 60.41 ± 4.35 | 52.57 ± 4.65 | **78.38 ± 4.78** | 58.24 ± 4.28 | 69.05 ± 2.15 | 77.70 ± 3.34 |
| Model A | Four-class | Macro-F1 | 60.10 ± 4.73 | 52.63 ± 4.37 | **78.28 ± 4.85** | 57.75 ± 4.62 | 68.84 ± 2.15 | 77.65 ± 3.39 |
| Model B | Stage 1 | ACC | 78.35 ± 1.72 | 80.58 ± 2.99 | 93.42 ± 1.86 | 78.24 ± 1.84 | 91.96 ± 1.64 | **93.64 ± 1.60** |
| Model B | Stage 1 | AUC | 86.31 ± 1.60 | 84.02 ± 2.82 | 98.70 ± 0.66 | 86.27 ± 1.61 | 96.83 ± 0.68 | **98.78 ± 0.53** |
| Model B | Stage 1 | Macro Recall | 78.34 ± 1.71 | 80.58 ± 2.99 | 93.42 ± 1.87 | 78.23 ± 1.85 | 91.95 ± 1.67 | **93.64 ± 1.60** |
| Model B | Stage 1 | Macro-F1 | 78.33 ± 1.71 | 80.57 ± 2.98 | 93.41 ± 1.87 | 78.19 ± 1.86 | 91.93 ± 1.67 | **93.64 ± 1.60** |
| Model B | Stage 2 | ACC | 68.47 ± 3.91 | 66.49 ± 6.20 | 81.80 ± 1.75 | 67.93 ± 6.41 | 78.56 ± 2.70 | **85.05 ± 3.15** |
| Model B | Stage 2 | Macro-F1 | 68.22 ± 3.73 | 65.83 ± 6.33 | 81.78 ± 1.70 | 67.53 ± 6.42 | 78.38 ± 2.73 | **85.00 ± 3.23** |
| Model C | Stage 1 | ACC | 78.35 ± 1.72 | 80.58 ± 2.99 | 93.42 ± 1.86 | 78.24 ± 1.84 | 91.96 ± 1.64 | **93.64 ± 1.60** |
| Model C | Stage 1 | AUC | 86.31 ± 1.60 | 84.02 ± 2.82 | 98.70 ± 0.66 | 86.27 ± 1.61 | 96.83 ± 0.68 | **98.78 ± 0.53** |
| Model C | Stage 1 | Macro Recall | 78.34 ± 1.71 | 80.58 ± 2.99 | 93.42 ± 1.87 | 78.23 ± 1.85 | 91.95 ± 1.67 | **93.64 ± 1.60** |
| Model C | Stage 1 | Macro-F1 | 78.33 ± 1.71 | 80.57 ± 2.98 | 93.41 ± 1.87 | 78.19 ± 1.86 | 91.93 ± 1.67 | **93.64 ± 1.60** |
| Model C | Stage 2 | ACC | 85.40 ± 4.56 | 83.06 ± 1.92 | **95.59 ± 1.42** | 85.13 ± 3.81 | 93.94 ± 2.19 | 94.90 ± 2.53 |
| Model C | Stage 2 | AUC | 92.62 ± 2.30 | 88.12 ± 1.14 | **99.17 ± 0.44** | 92.55 ± 2.02 | 98.20 ± 0.92 | 99.08 ± 0.47 |
| Model C | Stage 2 | Macro-F1 | 85.40 ± 4.56 | 83.03 ± 1.95 | **95.59 ± 1.43** | 85.12 ± 3.81 | 93.93 ± 2.20 | 94.90 ± 2.53 |
| Model C | Stage 3 | ACC | 71.08 ± 3.03 | 67.03 ± 5.38 | 83.24 ± 3.15 | 70.54 ± 3.35 | 80.54 ± 3.03 | **86.49 ± 3.52** |
| Model C | Stage 3 | AUC | 80.63 ± 3.55 | 68.85 ± 5.63 | 92.24 ± 2.81 | 80.41 ± 4.05 | 89.01 ± 2.60 | **92.68 ± 3.27** |
| Model C | Stage 3 | Macro Recall | 71.08 ± 3.03 | 67.03 ± 5.38 | 83.24 ± 3.15 | 70.54 ± 3.35 | 80.54 ± 3.03 | **86.49 ± 3.52** |
| Model C | Stage 3 | Macro-F1 | 70.87 ± 3.17 | 66.93 ± 5.45 | 83.23 ± 3.14 | 70.39 ± 3.40 | 80.52 ± 3.04 | **86.47 ± 3.54** |

Table S4. Class-wise calibration performance of Model B on the internal held-out test cohort.

| **Task** | **Class** | **Support** | **OvR Brier score** | **OvR ECE** |
| --- | --- | --- | --- | --- |
| Stage 1 | ICL | 37 | 0.0732 | 0.0683 |
| Stage 1 | Laser | 116 | 0.0732 | 0.0683 |
| Stage 2 | SMILE | 40 | 0.0937 | 0.0654 |
| Stage 2 | SURFACE | 23 | 0.0729 | 0.0655 |
| Stage 2 | LASIK | 53 | 0.1057 | 0.0823 |
| End-to-end | ICL | 37 | 0.0732 | 0.0683 |
| End-to-end | SMILE | 40 | 0.0764 | 0.0546 |
| End-to-end | SURFACE | 23 | 0.0633 | 0.0568 |
| End-to-end | LASIK | 53 | 0.1090 | 0.0779 |

Table S5. Algorithm-specific patient-grouped cross-validation performance of structured feature-set ablation models.

Table S5A. Stage 1 performance of structured feature-set ablation models

| **Model** | **Metric** | **LR** | **DT** | **RF** | **SVM** | **XGBoost** | **MLP** |
| --- | --- | --- | --- | --- | --- | --- | --- |
| M1 | ACC | 69.09 ± 1.75 | 74.66 ± 3.65 | 84.82 ± 3.02 | 69.09 ± 2.07 | 84.71 ± 1.92 | 72.32 ± 2.73 |
| M1 | AUC | 76.08 ± 1.64 | 79.39 ± 2.80 | 92.78 ± 2.09 | 76.08 ± 1.71 | 92.34 ± 1.34 | 81.35 ± 1.98 |
| M1 | Recall | 68.96 ± 4.39 | 70.52 ± 3.49 | 84.82 ± 2.88 | 69.41 ± 4.58 | 85.27 ± 1.42 | 72.99 ± 3.12 |
| M1 | F1-score | 69.00 ± 2.15 | 73.57 ± 3.78 | 84.83 ± 2.99 | 69.14 ± 2.32 | 84.81 ± 1.74 | 72.54 ± 1.90 |
| M2 | ACC | 76.46 ± 3.05 | 81.03 ± 2.58 | 93.53 ± 2.79 | 76.01 ± 2.61 | 94.19 ± 1.16 | 87.95 ± 1.29 |
| M2 | AUC | 83.96 ± 2.44 | 84.45 ± 3.42 | 98.68 ± 0.67 | 83.81 ± 2.64 | 98.49 ± 0.45 | 94.68 ± 1.25 |
| M2 | Recall | 74.10 ± 3.95 | 79.46 ± 5.11 | 90.84 ± 4.33 | 72.98 ± 3.80 | 92.63 ± 1.98 | 82.80 ± 2.65 |
| M2 | F1-score | 78.80 ± 2.88 | 80.65 ± 3.10 | 93.30 ± 2.98 | 75.23 ± 2.91 | 94.09 ± 1.25 | 87.27 ± 1.54 |
| M3 | ACC | 75.78 ± 3.31 | 81.25 ± 2.91 | 92.86 ± 2.52 | 76.34 ± 3.22 | 93.30 ± 2.58 | 89.40 ± 0.81 |
| M3 | AUC | 85.70 ± 1.60 | 86.00 ± 2.17 | 98.67 ± 0.67 | 85.77 ± 1.55 | 98.49 ± 0.77 | 95.69 ± 0.49 |
| M3 | Recall | 74.11 ± 4.47 | 78.35 ± 5.38 | 89.50 ± 3.39 | 74.10 ± 4.21 | 90.38 ± 4.93 | 84.59 ± 1.86 |
| M3 | F1-score | 75.33 ± 3.63 | 80.62 ± 3.33 | 92.59 ± 2.66 | 75.76 ± 3.48 | 93.02 ± 2.90 | 88.85 ± 0.92 |
| M4 | ACC | 76.01 ± 2.28 | 81.25 ± 1.87 | 93.86 ± 1.86 | 76.23 ± 3.02 | 93.75 ± 1.28 | 88.84 ± 1.32 |
| M4 | AUC | 83.84 ± 2.59 | 85.15 ± 2.02 | 98.77 ± 0.73 | 83.87 ± 2.86 | 98.51 ± 0.80 | 95.58 ± 1.12 |
| M4 | Recall | 74.32 ± 4.21 | 78.81 ± 3.98 | 92.63 ± 2.20 | 73.88 ± 4.58 | 93.31 ± 1.21 | 83.70 ± 2.73 |
| M4 | F1-score | 75.54 ± 2.78 | 80.76 ± 2.01 | 93.78 ± 1.91 | 75.60 ± 3.48 | 93.73 ± 1.27 | 88.21 ± 1.58 |
| M5 | ACC | 77.68 ± 1.66 | 79.58 ± 2.26 | 93.75 ± 1.54 | 77.57 ± 1.89 | 94.42 ± 1.36 | 90.96 ± 1.22 |
| M5 | AUC | 85.85 ± 1.36 | 84.00 ± 1.86 | 98.80 ± 0.69 | 85.82 ± 1.29 | 98.68 ± 0.61 | 96.32 ± 0.45 |
| M5 | Recall | 75.68 ± 4.21 | 76.12 ± 3.72 | 90.40 ± 2.87 | 74.10 ± 4.64 | 91.96 ± 1.82 | 86.37 ± 1.99 |
| M5 | F1-score | 77.17 ± 2.25 | 78.83 ± 2.43 | 93.51 ± 1.70 | 76.69 ± 2.50 | 94.28 ± 1.40 | 90.54 ± 1.17 |
| M6 | ACC | 78.35 ± 1.72 | 80.58 ± 2.99 | 93.42 ± 1.86 | 78.24 ± 1.84 | 93.64 ± 1.60 | 91.96 ± 1.64 |
| M6 | AUC | 86.31 ± 1.60 | 84.02 ± 2.82 | 98.70 ± 0.66 | 86.27 ± 1.61 | 98.78 ± 0.53 | 96.83 ± 0.68 |
| M6 | Recall | 76.56 ± 3.48 | 79.25 ± 2.44 | 90.85 ± 2.26 | 74.99 ± 4.21 | 91.74 ± 1.16 | 87.25 ± 4.46 |
| M6 | F1-score | 77.92 ± 2.03 | 80.35 ± 2.74 | 93.24 ± 1.95 | 77.45 ± 2.39 | 93.53 ± 1.58 | 91.50 ± 2.01 |

Table S5B. Stage 2 performance of structured feature-set ablation models

| **Model** | **Metric** | **LR** | **DT** | **RF** | **SVM** | **XGBoost** | **MLP** |
| --- | --- | --- | --- | --- | --- | --- | --- |
| M1 | ACC | 56.58 ± 2.76 | 62.70 ± 5.05 | 71.53 ± 3.54 | 58.02 ± 1.67 | 71.71 ± 3.10 | 59.64 ± 4.20 |
| M1 | Macro-F1 | 54.97 ± 2.36 | 62.38 ± 5.16 | 71.27 ± 3.89 | 54.30 ± 2.17 | 71.43 ± 3.24 | 58.42 ± 4.70 |
| M2 | ACC | 67.75 ± 5.65 | 66.85 ± 2.81 | 81.98 ± 3.74 | 68.47 ± 4.56 | 82.52 ± 4.01 | 74.41 ± 3.67 |
| M2 | Macro-F1 | 67.24 ± 5.40 | 66.56 ± 2.64 | 81.91 ± 3.78 | 68.02 ± 4.32 | 82.44 ± 4.11 | 74.06 ± 3.72 |
| M3 | ACC | 70.63 ± 4.05 | 67.03 ± 4.51 | 82.70 ± 3.67 | 69.01 ± 4.92 | 81.62 ± 2.18 | 76.58 ± 0.81 |
| M3 | Macro-F1 | 70.40 ± 3.98 | 66.80 ± 4.49 | 82.69 ± 3.60 | 68.68 ± 4.65 | 81.55 ± 2.20 | 76.18 ± 0.86 |
| M4 | ACC | 68.65 ± 7.15 | 69.91 ± 1.77 | 82.16 ± 1.75 | 67.93 ± 6.61 | 82.88 ± 3.74 | 74.23 ± 2.52 |
| M4 | Macro-F1 | 68.26 ± 7.19 | 69.85 ± 1.72 | 82.11 ± 1.65 | 67.54 ± 6.66 | 82.88 ± 3.78 | 73.92 ± 2.30 |
| M5 | ACC | 67.57 ± 4.03 | 68.11 ± 2.52 | 82.52 ± 4.05 | 68.11 ± 4.17 | 83.96 ± 3.71 | 75.32 ± 3.63 |
| M5 | Macro-F1 | 67.30 ± 3.77 | 67.86 ± 2.33 | 82.46 ± 4.05 | 67.80 ± 4.03 | 83.95 ± 3.76 | 75.00 ± 3.61 |
| M6 | ACC | 68.47 ± 3.91 | 66.49 ± 6.20 | 81.80 ± 1.75 | 67.93 ± 6.41 | 85.05 ± 3.15 | 78.56 ± 2.70 |
| M6 | Macro-F1 | 68.22 ± 3.73 | 65.83 ± 6.33 | 81.78 ± 1.70 | 67.53 ± 6.42 | 85.00 ± 3.23 | 78.38 ± 2.73 |

Table S6. Hyperparameter grids for Stage 1 classifiers.

| **Algorithm** | **Hyperparameter grid** |
| --- | --- |
| Logistic regression | C: [0.01, 0.05, 0.1, 0.2, 1, 5, 10]; class_weight: [None, "balanced"] |
| Decision tree | max_depth: [None, 2, 3, 5, 7]; min_samples_split: [2, 5, 10]; min_samples_leaf: [1, 2, 5]; class_weight: [None, "balanced"] |
| Random forest | n_estimators: [200, 500]; max_depth: [None, 5, 10]; min_samples_leaf: [1, 2, 5]; max_features: ["sqrt", "log2"]; class_weight: [None, "balanced"] |
| SVM | C: [0.01, 0.05, 0.1, 1, 5, 10]; class_weight: [None, "balanced"] |
| XGBoost | n_estimators: [200, 300, 500]; max_depth: [2, 3, 5]; learning_rate: [0.03, 0.05, 0.07]; subsample: [0.7, 0.8, 0.9]; colsample_bytree: [0.7, 0.9]; colsample_bylevel: [0.7, 1.0] |
| MLP | hidden_size: [32, 64, 128]; lr:[0.001, 0.0005]; weight_decay: [0, 1e-4]; dropout: [0.0, 0.1]; epochs: [300] |

Table S7. Hyperparameter grids for Stage 2 classifiers.

| **Algorithm** | **Hyperparameter grid** |
| --- | --- |
| Logistic regression | C: [0.01, 0.1, 0.2, 1, 10]; class_weight: [None, "balanced"] |
| Decision tree | max_depth: [None, 3, 5, 7]; min_samples_split: [2, 5, 10]; min_samples_leaf: [1, 2, 5]; class_weight: [None, "balanced"] |
| Random forest | n_estimators: [200, 500]; max_depth: [None, 5, 10]; min_samples_leaf: [1, 2]; max_features: ["sqrt", "log2"]; class_weight: [None, "balanced"] |
| SVM | C: [0.01, 0.1, 1, 10]; class_weight: [None, "balanced"] |
| XGBoost | n_estimators: [200, 500]; max_depth: [2, 3, 4]; learning_rate: [0.03, 0.05, 0.09]; subsample: [0.8, 0.9]; colsample_bytree: [0.6, 0.8] |
| MLP | hidden_size: [32, 64, 128]; lr:[0.001, 0.0005]; weight_decay: [0, 1e-4]; epochs: [300] |

Table S8. Final selected hyperparameters for Model B classifiers.

| **Stage** | **Algorithm** | **Final selected hyperparameters** |
| --- | --- | --- |
| Stage 1: ICL vs laser | Logistic regression | C = 0.1; class_weight = None |
| Stage 1: ICL vs laser | Decision tree | class_weight = "balanced"; max_depth = 7; min_samples_leaf = 5; min_samples_split = 2 |
| Stage 1: ICL vs laser | Random forest | class_weight = None; max_depth = None; max_features = "log2"; min_samples_leaf = 1; n_estimators = 500 |
| Stage 1: ICL vs laser | SVM | C = 0.01; class_weight = "balanced" |
| Stage 1: ICL vs laser | XGBoost | colsample_bylevel = 0.7; colsample_bytree = 0.9; learning_rate = 0.05; max_depth = 5; n_estimators = 300; subsample = 0.7 |
| Stage 1: ICL vs laser | MLP | hidden_size = 64; lr = 0.001; weight_decay = 0.0; dropout = 0.0; epochs = 300 |
| Stage 2: SMILE vs SURFACE vs LASIK | Logistic regression | C = 0.1; class_weight = None |
| Stage 2: SMILE vs SURFACE vs LASIK | Decision tree | class_weight = None; max_depth = 5; min_samples_leaf = 1; min_samples_split = 2 |
| Stage 2: SMILE vs SURFACE vs LASIK | Random forest | class_weight = None; max_depth = None; max_features = "sqrt"; min_samples_leaf = 1; n_estimators = 500 |
| Stage 2: SMILE vs SURFACE vs LASIK | SVM | C = 0.01; class_weight = None |
| Stage 2: SMILE vs SURFACE vs LASIK | XGBoost | colsample_bytree = 0.6; learning_rate = 0.05; max_depth = 4; n_estimators = 500; subsample = 0.9 |
| Stage 2: SMILE vs SURFACE vs LASIK | MLP | hidden_size = 64; lr = 0.001; weight_decay = 0.0; epochs = 300 |

Table S9. Strongest Spearman correlations between pure Corvis ST dynamic response/stiffness parameters and tomographic, pachymetric, deviation/risk, and composite risk-related variables.

| **Pure Corvis ST parameter** | **Rank** | **Compared variable** | **Variable domain** | **Spearman ρ** | **Absolute ρ** | **FDR-adjusted P value** | **Valid pairs** |
| --- | --- | --- | --- | --- | --- | --- | --- |
| DA-Ratio | 1 | Dt | Pentacam-derived deviation/risk index | 0.618 | 0.618 | <0.001 | 763 |
| DA-Ratio | 2 | TCT | Pentacam tomography/pachymetry/anterior segment | -0.617 | 0.617 | <0.001 | 763 |
| DA-Ratio | 3 | CBI | Pressure-/thickness-profile-/composite risk index | 0.563 | 0.563 | <0.001 | 763 |
| IR | 1 | TCT | Pentacam tomography/pachymetry/anterior segment | -0.521 | 0.521 | <0.001 | 763 |
| IR | 2 | Dt | Pentacam-derived deviation/risk index | 0.521 | 0.521 | <0.001 | 763 |
| IR | 3 | Da | Pentacam-derived deviation/risk index | 0.498 | 0.498 | <0.001 | 763 |
| SP-A1 | 1 | Dt | Pentacam-derived deviation/risk index | -0.731 | 0.731 | <0.001 | 763 |
| SP-A1 | 2 | TCT | Pentacam tomography/pachymetry/anterior segment | 0.731 | 0.731 | <0.001 | 763 |
| SP-A1 | 3 | CBI | Pressure-/thickness-profile-/composite risk index | -0.627 | 0.627 | <0.001 | 763 |
| SSI | 1 | bIOP | Pressure-/thickness-profile-/composite risk index | 0.349 | 0.349 | <0.001 | 763 |
| SSI | 2 | Db | Pentacam-derived deviation/risk index | 0.241 | 0.241 | <0.001 | 763 |
| SSI | 3 | Front K max | Pentacam tomography/pachymetry/anterior segment | -0.232 | 0.232 | <0.001 | 763 |

Figure S1. ROC curves of the six classification algorithms used in the two stages of Model B.

footnote: (A) ROC curves for Stage 1 of Model B. (B) Macro-ROC curves for Stage 2 of Model B. Classes 0, 1, and 2 represent SMILE, SURFACE, and LASIK, respectively.

Figure S2. Calibration curves of the selected Model B.

footnote: (A) End-to-end four-class calibration curve. (B) Stage 1 ICL versus laser calibration curve. (C) Stage 2 SMILE versus SURFACE versus LASIK calibration curve.

Figure S3. Spearman correlation heatmap between pure Corvis ST dynamic response/stiffness parameters and tomographic, pachymetric, deviation/risk, and composite risk-related variables.

footnote: (A) Corvis parameters vs Tomographic, keratometric, pachymetric, and anterior-segment variables; (B) Corvis parameters vs Deviation/risk and pressure-/thickness-profile-/composite variables.

Algorithm S1 Patient-level model development and evaluation workflow
